# A deep convolutional neural network approach for astrocyte detection

**DOI:** 10.1038/s41598-018-31284-x

**Published:** 2018-08-27

**Authors:** Ilida Suleymanova, Tamas Balassa, Sushil Tripathi, Csaba Molnar, Mart Saarma, Yulia Sidorova, Peter Horvath

**Affiliations:** 1 Laboratory of Molecular Neuroscience, Research Program in Developmental Biology, Institute of Biotechnology (HiLIFE), University of Helsinki, Viikinkaari 5D, FI-00014 Helsinki, Finland; 2 Synthetic and Systems Biology Unit, Hungarian Academy of Sciences, Biological Research Centre (BRC), Temesvári körút 62, 6726 Szeged, Hungary; 3 Research Program Unit, Helsinki Institute of Life Science (HiLIFE), Faculty of Medicine, University of Helsinki, Haartmaninkatu 8, 00014 Helsinki, Finland; 4 Institute for Molecular Medicine Finland (FIMM), University of Helsinki, Tukholmankatu 8, 00014 Helsinki, Finland

## Abstract

Astrocytes are involved in various brain pathologies including trauma, stroke, neurodegenerative disorders such as Alzheimer’s and Parkinson’s diseases, or chronic pain. Determining cell density in a complex tissue environment in microscopy images and elucidating the temporal characteristics of morphological and biochemical changes is essential to understand the role of astrocytes in physiological and pathological conditions. Nowadays, manual stereological cell counting or semi-automatic segmentation techniques are widely used for the quantitative analysis of microscopy images. Detecting astrocytes automatically is a highly challenging computational task, for which we currently lack efficient image analysis tools. We have developed a fast and fully automated software that assesses the number of astrocytes using Deep Convolutional Neural Networks (DCNN). The method highly outperforms state-of-the-art image analysis and machine learning methods and provides precision comparable to those of human experts. Additionally, the runtime of cell detection is significantly less than that of other three computational methods analysed, and it is faster than human observers by orders of magnitude. We applied our DCNN-based method to examine the number of astrocytes in different brain regions of rats with opioid-induced hyperalgesia/tolerance (OIH/OIT), as morphine tolerance is believed to activate glia. We have demonstrated a strong positive correlation between manual and DCNN-based quantification of astrocytes in rat brain.

## Introduction

Astrocytes are a type of glial cells within the central nervous system (CNS). On average, there are 67–86 billion neurons and 40–85 billion glial cells in human brain^1^. The proportion of astrocytes in the CNS varies by brain and spinal cord regions, and it is estimated to range from 20% to 40% of all glia^2^. Astrocytes respond to injury and CNS diseases, and play an important role in the development and maintenance of chronic pain and certain psychiatric disorders such as autism spectrum disorders and schizophrenia^3,4^.

Morphologically, astrocytes are star-shaped structures with small rounded bodies, and numerous long, ramified branches. These cells are disseminated distinctly over the nervous tissue. Investigations of morphological changes of astrocytes in pathological conditions or after chemical/genetic perturbations is usually carried out through the rigorous process of cell staining, imaging, image analysis and quantification. The versatility of the branching structure of astrocytes makes their automatic detection rather challenging. Therefore, developing fully automated counting methods applicable to immunohistochemically stained images that require no user intervention is a major issue. Traditionally, manual or semi-automated techniques have been used to evaluate the number of CNS cells in samples of interest. However, manual counting processes are time-intensive, cumbersome and prone to human errors. Commonly used open-source tools for general cell quantification such as ImageJ^5^, custom scripts or ilastik^6^ have technical limitations concerning the accurate detection of astrocytes because of their complex morphology^2,7,8^. These tools are based on either machine-learning methods (ilastik) or thresholding (ImageJ, custom scripts).

Currently, no efficient image analysis tools are available to quantify astrocytes from large-scale histology datasets. A novel potential way to circumvent manual or semi-automated techniques is to use Deep Convolutional Neural Network (DCNN) approaches to identify cells. Deep learning is a type of machine learning approach based on learning from multiple layers of feature extraction, and can be used to analyse complex data such as images, sounds and texts^9–14^. Recently, DCNN has gained attention in the field of computational cell biology^15–19^ and has shown remarkable success in complex image classification tasks^20^.

Here, we propose a DCNN-based method and an open-source software platform enabling biologists and pathologists to accurately detect astrocytes in immunohistological images (Supplementary Software 1). Our software, *FindMyCells* (www.findmycells.org), is a fully automated and user-friendly software for a highly precise detection of cells in microscopy images. We remark, however, that the software can also be used for a range of other object detection tasks if the underlying DCNN module is properly trained to recognize the objects of interest. DCNN shows a tremendous improvement in accuracy in terms of detecting complex morphologies such as astrocytes. Based on the results from our analyses, FindMyCells strongly outperforms other computational methods and is comparable to the performance of human experts.

To validate the proposed software, we compared the cell counts produced by human experts and FindMyCells, as well as by three other software tools (ilastik, custom threshold-based script^21^, ImageJ), analysing brain tissues of rats treated with repeated injections of morphine to induce opioid-induced hyperalgesia/tolerance (OIH/OIT). OIH/OIT is a common complication of prolonged opioid therapy which is characterized by enhanced pain and/or lack of pain relief ^22^. Recent evidence supports the role of spinal glial activation in the development and maintenance of OIH/OIT^23,24^, but the implications of brain glia in this process needs to be clarified. Therefore, utilizing immunohistochemistry (IHC) we analysed glial cells from different brain regions that are believed to be involved in the generation and maintenance of pain^25,26^. The model we applied, including the exact biological experiments and outcomes are described in detail by Jokinen and co-authors^27^. In the current study, we first counted astrocytes by FindMyCell, ilastik, custom threshold-based script^21^ and ImageJ in randomly selected IHC stained sections from the brains of control (CTR) and morphine-treated animals described by Jokinen^27^. We compared the performance of FindMyCells to that of semi-automated tools based on machine-learning principles or manual thresholding. To assess the applicability of FindMyCells to the real laboratory tasks, we selected images of the striatum where we had previously observed changes in the number of astrocytes^27^. We analysed the microscopy images of these samples by both manual counting and FindMyCells, separately, and assessed the correlation between FindMyCell’s output and the manual counting of astrocytes.

## Materials and Methods

### Preparation of samples and data sets for analysis

**Figure 1:**
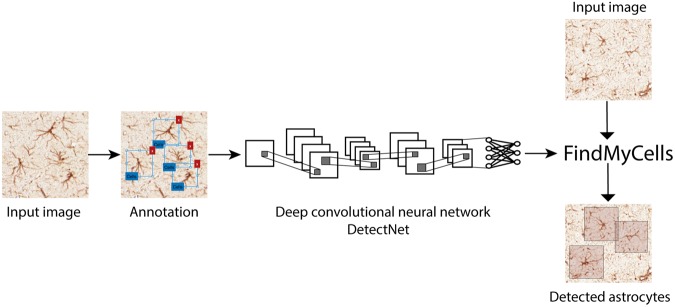
Schematic overview of the proposed framework and the FindMyCells software based on DCNN. Left: 15,000 cells were annotated with their bounding boxes, and a DCNN method was trained based on the DetectNet architecture for object detection^37^. Right: The FindMyCells software starts out from the raw image data. A DCNN model receives the raw images as input, and after prediction it marks the identified astrocytes with bounding boxes around the cell body. The software also exports the coordinates of detected cells into a table (not shown on the scheme).

We used an experimental data set containing microscopy images of several different regions of rat brain to train and examine the performance of the proposed DCNN approach. Samples were treated as described by Jokinen and co-authors^27^. The experimental work was conducted according to the guidelines of local authorities and the International Association for the Study of Pain^28^, and in line with the European Communities Council Directive of 24 November 1986. The study protocol was approved by the provincial government of Southern Finland (Uudenmaan Lääninhallitus, Hämeenlinna, Finland). Briefly, brains of rats with and without OIH/OIT were sectioned, and astrocytes were labelled with glial fibrillary acidic protein (GFAP) antibodies (Cat#G-3893, Sigma-Aldrich, St. Louis, MO, USA), followed by staining using a VECTASTAIN ABC HRP Kit (Cat#PK-4002 Vector Laboratories, Burlingame, CA, USA). Slides were scanned using the 3DHISTECH Scanner (3DHISTECH Ltd., Budapest, Hungary). The images for analysis were saved in tiff format from the Pannoramic Viewer software using a 20x magnification. Two data sets were prepared: the training data set contained 1200 images of random regions of rat brains, and the validation data set contained 12 images of the striatum. Images in the training data set were handled as shown in Fig. 1. Briefly, after sample preparation, astrocytes were annotated and a deep neural network was trained. Next, DCNN prediction was executed, and cell count and coordinates were collected. Details of these procedures are described below.

### Annotation procedure

Annotations were made manually using the Matlab region of interest (ROI) Training Image Labeler (TIL) application. Cells were inserted into the training set by drawing a bounding rectangle around them, executed by the expert. Rectangles were allowed to intersect each other and their size varied. TIL provided a comfortable interface to precisely adjust bounding boxes. Based on our experience, the best results were achieved by keeping bounding boxes tight to the cells. The final training set was labelled by a single expert; constitutes a total of 1120 images and 15,000 cells thus providing a rich platform for testing the accuracy of astrocyte detection (Supplementary data 1 and BBBC0xx. The annotations and the image data were deposited to the Broad Bioimage Benchmark Collection).

For performance evaluation, another expert was asked to label a smaller test set of ~130 different images containing ~2200 cells. This validation data set was annotated in duplicate at two different time points by both of the experts to characterize inter- and intra-observer accuracy.

### Software platform and convolutional neural networks

The proposed new tool for astrocyte detection was implemented in Python 2.7+. We developed the software’s graphical interface using the PyQt5 package. For the deep learning implementation, we used the DetectNet (https://devblogs.nvidia.com/parallelforall/detectnet-deep-neural-network-object-detection-digits/) architecture which is an extension of the *caffe* package^29^.

The architecture of DetectNet was specified in a *caffe* model definition file and it is composed of five main parts: (1) data ingest and augmentation, (2) GoogLeNet^30^ fully connected network (FCN), (3) loss function measurement, (4) bounding box clustering and (5) mean average precision (mAP) calculation. While parts 1–3 belong to the training section, the parts 4–5 are for result validation. Here we give a short description of the DetectNet architecture.

In the first part, input data characteristics, such as training patch size or data augmentation details can be set. To achieve a precise and sensitive object detection, comprehensive data augmentation is fundamental. Augmentation serves to assure that the training algorithm considers the same object only once. A main advantage of using this technique is evading overfitting.

The fully connected network configuration of DetectNet is basically the same as that of GoogLeNet, with the difference that the input, the last pooling and the output layers are removed. The benefit of DetectNet’s using the same structure is the applicability of the GoogLeNet-based pre-trained model, which significantly increases the efficiency of the training process.

As for part 3 of DetectNet, two loss functions are linearly combined for general optimization. One of them measures the diversity of the true and predicted object hit in a training data sample, while the other one computes the mean absolute difference for the true and predicted corners of the bounding boxes. By applying *caffe*’s functions, these loss values are minimized and a summary can be obtained, which is the final objective of the training process. To get a real-time feedback during training about the quality of the model, different metrics were applied on the validation set. The most important metric was mAP. It is an output rate calculated by DetectNet from the values of precision and recall.

The NVIDIA DIGITS (https://github.com/NVIDIA/DIGITS) framework was used to train the network. We used the ADAM (Adaptive Moment Estimation)^31^ solver with 0.0001 as the base learning rate, with a ‘step down’ learning rate policy, applying a 33% step size and 0.1 gamma. ADAM is a method that computes adaptive learning rates for each parameter. To significantly speed up our training process, we used the pre-trained weights derived from an ImageNet-trained GoogLeNet. Next, training computations were conducted on a notebook PC with a 2.3 GHz Intel Core i5, 8GB RAM memory and an NVIDIA GeForce GTX. The software was then run on a 3.5 GHz Intel Core i7-4770K computer with 16GB memory and an NVIDIA Titan Xp graphic card.

### Evaluation methods and metrics

To evaluate the performance of the proposed framework we measured precision, recall and F1 score provided by two different annotators and four different computational methods. For the quantitative characterization of these parameters we made an object matching between the ground truth objects and those of the detection of interest. For matching objects, we used the Hungarian method that provides optimal pairing between point sets. To calculate this matching, we defined pairing as an assignment problem. We assigned a weight to each pair of ground truth objects and detected matching objects. The assigned weight was set to infinite when the objects in a pair had no overlap. Otherwise the weight was set to the reciprocal of the area of overlap. The total cost of pairing was defined as the sum of all the weights of paired objects^32^.

A correct detection (true positive, TP) was established when a ground truth object had a matched pair. False positive (FP) detection was quantified when an extra object was present in the applied method’s output, while a false negative (FN) was considered in case of missing objects. Based on these aspects, detection accuracy, precision (P) = TP/(TP + FP), recall (R) = TP/(TP + FN), and F1 score = 2*P*R/(P + R) statistics were calculated.

We compared a total of six sets of astrocyte detection data, of which two were generated by human experts, one by the FindMyCells pipeline, one by ilastik, one by thresholding and simple morphology operations^32^, and one by using standard ImageJ operations. To measure the self- and cross-accuracies of human experts, the same test images were blindly presented to the annotators for re-labelling. To minimize bias, duplicate images were mirrored and/or rotated. A total of ~30 different images containing ~300 cells were manually annotated using the Matlab ROI Training Image Labeler application.

In ilastik, we used pixel and object classifications with Gaussian Smoothing colour/intensity feature, Gaussian Gradient Magnitude and Difference of Gaussians edge features. For the threshold-based method we chose an algorithm with manual thresholding offered by the Matlab software. The algorithm detects positive cells when the intensity of their staining is substantially higher than the background. All pixels outside the region of interest are set to zero. Matlab’s built-in functions trace the boundaries of cells/objects. Objects smaller than the average size of cells were excluded from the quantification by using an object size threshold, set manually^21^. For ImageJ, we used intermodes threshold, which has the substantial advantage of removing background noise and identifying individual cells in the image. Alike for the threshold-based method, objects smaller than the average size of cells were excluded.

## Results

**Figure 2:**
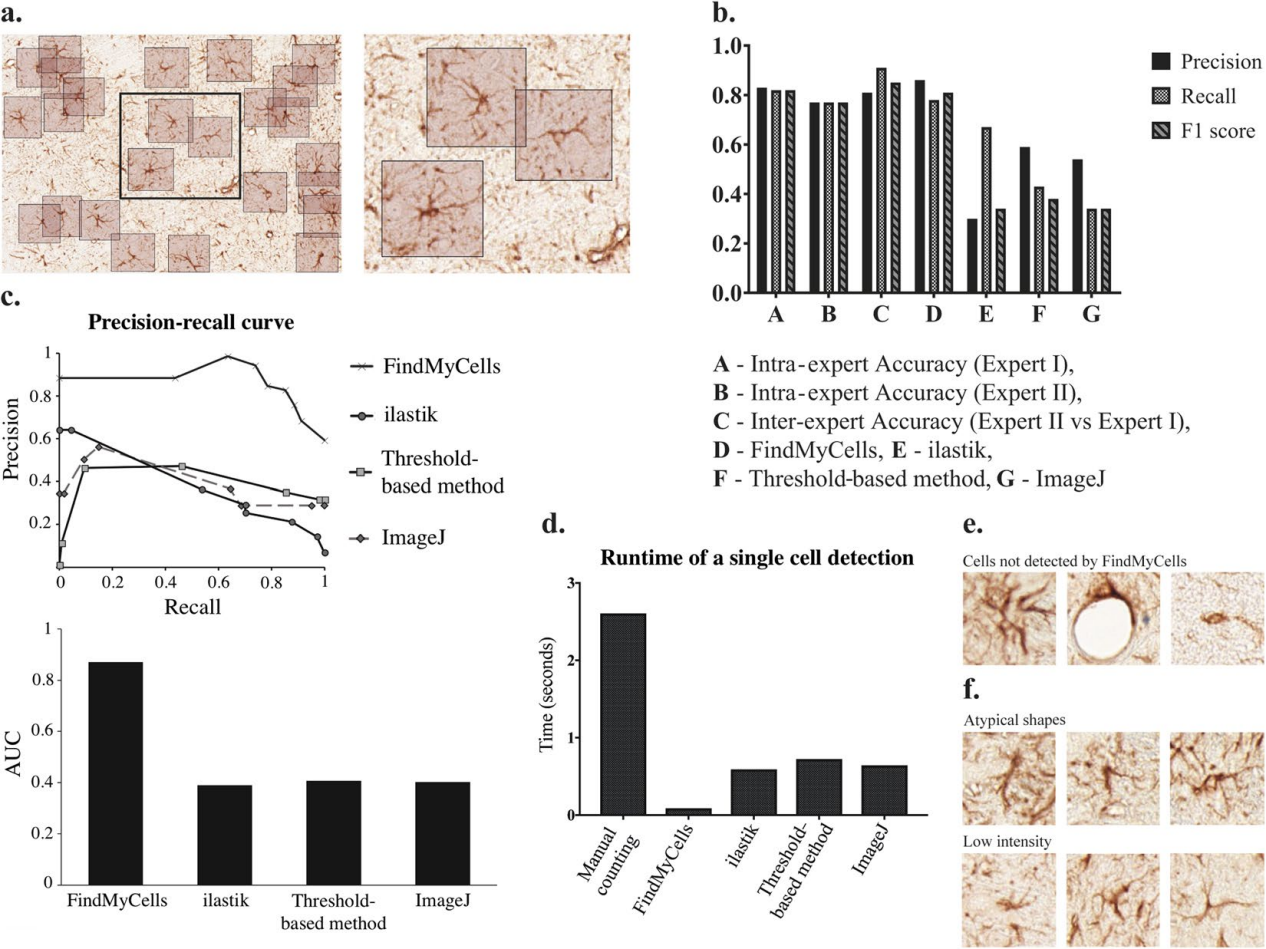
Examples of astrocyte detection by FindMyCells in midbrain samples of OIH/OIT and CTR rats. (**a**) Detection results. (**b**) Precision, recall and F1 score metrics for the human experts, FindMyCells, ilastik and the threshold-based algorithm. (**c**) Precision-recall curve and AUC values. (**d**) Average detection time for the human observer and for the computational methods. (**e**) Cases when FindMyCells fails. (**f**) Cases when FindMyCells and experts disagree.

We compared the performance of the two field experts and of the four computational methods for the quantification of astrocytes using precision, recall, and F1 metrics (Fig. 2) calculated as described in the ‘Methods’ section. Figure 2a shows examples of astrocyte detection in images using the FindMyCell software.

Figure 2b illustrates the comparison of the results for applying different quantification methods to image analysis. First, we measured the self-accuracy of human experts. As expected, astrocyte detection proved to be a complex issue, thus even intra-observer accuracy was far from being perfect. F1 score was 0.82 and 0.77 for the two human experts, respectively. We also evaluated inter-observer accuracy, for which the F1 score was 0.85. In each case the test set annotated by the first human expert was used as ground truth, and we remark that this same annotator labelled the training set of 15,000 cells.

We expected that an appropriate computational method would perform close to the inter-observer accuracy, and might approach the self-accuracy of our experts. Indeed, an outstanding performance was observed for the proposed DCNN. Its F1 score, precision and recall values were similar to the human experts’ self-accuracy (P = 0.86 R = 0.78, F1 = 0.81). We also tested ilastik (P = 0.30, R = 0.67, F1 = 0.34), the threshold-based analysis method (P = 0.59, R = 0.43, F1 = 0.38), and ImageJ (P = 0.54, R = 0.34, F1 = 0.34) for these parameters. These tools were highly outperformed by the human experts, as well as by the FindMyCells software.

We also generated a precision-recall (PR) curve and analysed the area under the curve (AUC). Figure 2c demonstrates that both the PR curve and the AUC for FindMyCells are twice as favourable as those for ilastik, the threshold-based method or ImageJ.

In practice, the runtime of any computational method is an important parameter concerning its performance. Therefore, we evaluated the average runtime of annotating a single cell by the different methods. For the human experts, it took approximately 2.6 seconds to annotate a single cell. The same value is approximately 0.088 seconds for FindMyCells, 0.59 seconds for ilastik, about 0.72 seconds for the threshold-based method and 0.64 seconds for ImageJ on the same computer (Fig. 2d), indicating that FindMyCells is characterized by a highly beneficial runtime besides its good-quality performance. It is important to remark that FindMyCells uses highly parallelized GPU calculations. A drawback of FindMyCells is that it required 15,000 annotated cells (~11 hours of continuous labelling) and a strong GPU for training the model (~18 hours of training time, 300 epochs). Statistics for all evaluated metrics are shown in Supplementary Table 1.

Regarding limitations, there are some instances when our tool failed to detect astrocytes correctly. These situations include cases when 1) the tool fails to detect astrocytes (missing recognition); 2) the tool detects extra astrocytes which the experts, either or none of them, did not detect as astrocytes (positive recognition); and 3) the experts disagree with the software in detecting an astrocyte (false positive recognition). We speculate that the possible reasons for any discrepancies in accuracy of the tool in detecting astrocytes can be associated with an atypical shape of the cells-of-interest or poor-staining or low-intensity signals of the sample. Figure 2e,f illustrate two of these situations.

**Figure 3:**
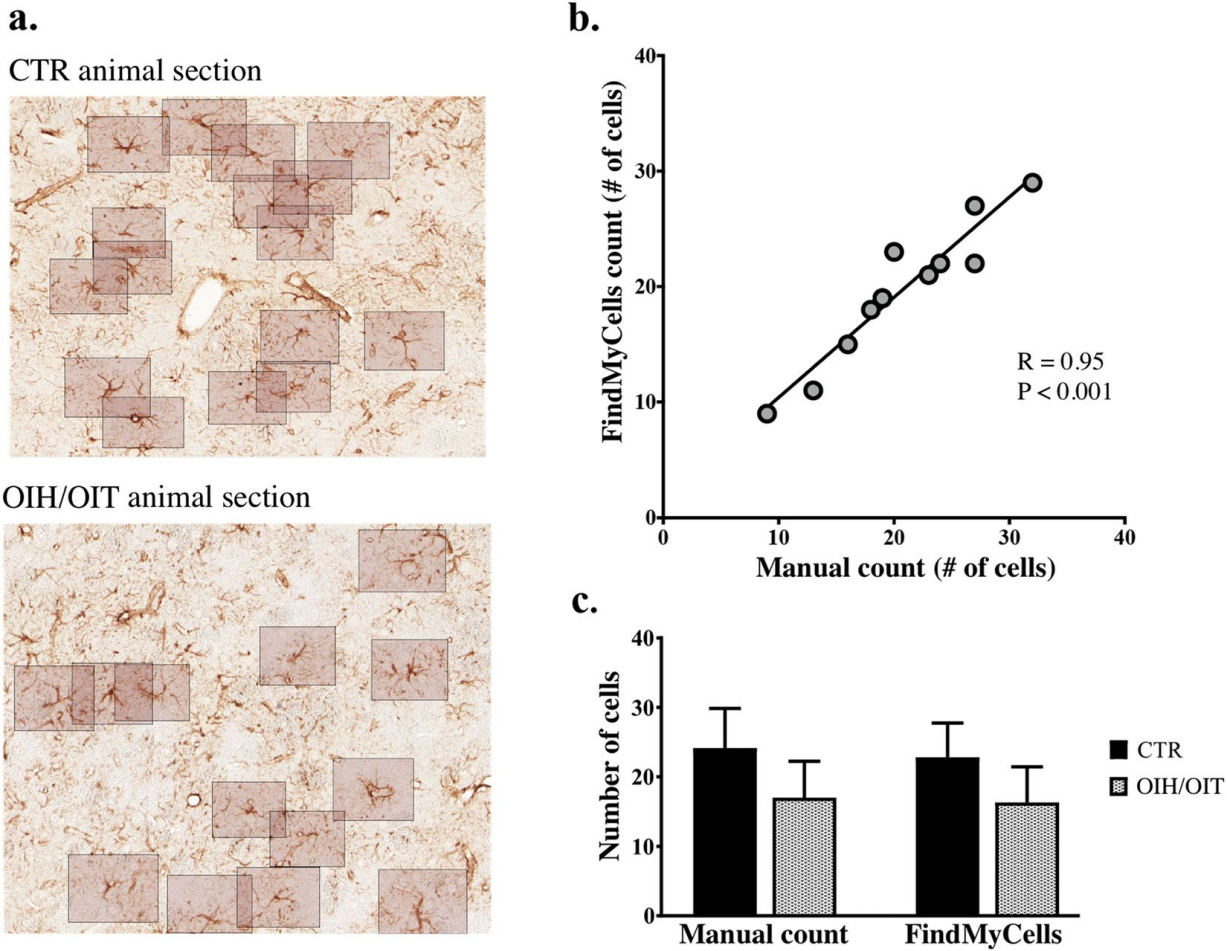
Examples of astrocyte detection in striatal samples of OIH/OIT and CTR rat groups. (**a**) Detection results. (**b**) Pearson correlation between FindMyCells output data and manual counting of astrocytes per image. (**c**) Number of astrocytes detected by human experts and FindMyCells.

Finally, we evaluated the proposed method on detecting astrocytes in a CTR and a drug-treated tissue environment, and compared its F1 score with that of the human experts. Twelve striatal sections of morphine treated and CTR animals (6 per group) were used for the experiment. Cells were counted manually and FindMyCells (Fig. 3a), respectively, and the cell count ratio for the treated vs. CTR groups was computed. The computed ratio was 0.703 for manual counting and 0.715 for FindMyCells, suggesting that our approach is precise and robust in counting cells, indicating an exceedingly high, ~99% measurement similarity. For each analysed image, results for the proposed method were consistent with those obtained by manual counting, with a Pearson correlation of R = 0.95 (Fig. 3b), indicating a highly strong correlation between FindMyCell’s output and the manual counting of astrocytes. The results for astrocyte quantification by FindMyCells are in line with previously published data^27^.

## Discussion

In this paper we introduced a novel, fully automated software FindMyCells (www.findmycells.org) for the accurate detection of astrocytes, a special type of neurons affected in various CNS pathologies, including trauma, stroke, neurodegenerative disorders or chronic pain. Astrocytes are characterized by a huge variation in appearance, size and shape which complicates their detection, although their precise and high-scale identification and analysis would be essential for a better understanding of their role in the aforementioned brain pathologies.

Manual counting is considered to be the gold standard and most widely used approach for astrocyte detection in the world^33–35^. However, the manual approach is prone to the operator’s subjectivity, and is associated with non-reproducible and imprecise experimental results. Furthermore, subjective bias may occur when the operator is not blinded to the sample.

We propose a DCNN-based method and an open-source software platform enabling biologists and pathologists to accurately detect astrocytes in immunohistological images. FindMyCells is a fully automated and user-friendly software for the precise detection of these cells in bright-field images. To train the DCNN module of our software presented in this paper, we labelled 15,000 cells manually and achieved and F1 score of 84%. The F1 score for the FindMyCells software might be further improved when more annotations are available. Nevertheless, even in its current state FindMyCells significantly outperforms semi-automated methods based on either machine-learning (ilastik) or classical image analysis principles, and has an F1 score comparable to that of human experts. A highly strong correlation was shown between FindMyCell’s output and the manual counting of astrocytes. Importantly, we detected a pronounced variation in the F1 score between the two human experts, although both of them work in the same lab with a similar domain knowledge. We consider that the fairly low level of intra-observer accuracy indicates the challenging nature of a reliable detection of astrocytes.

To validate the proposed system, we compared the human observers’ detection-based count with the performance of FindMyCells. Our results on real data show that the values for the ratio of astrocytes in the brains of morphine treated and CTR rats differ by only 1% when cell counting is executed by FindMyCells or by the human experts, suggesting that the proposed method is highly applicable to real tasks in everyday practice. Importantly, these results are in line with literature data^27^.

Applications of FindMyCells to other model organisms clearly warrant further studies. Many scientists use other mammalian (mice, dogs, etc.) and non-mammalian (fruit flies, zebrafish, worms, etc.) organisms in their research, and human samples are analysed in clinical practice. The morphology of glial cells in non-mammalian species may differ significantly from the morphology of rat astrocytes, and it is unlikely that the current DCNN would efficiently identify these cells in tissue samples from non-mammalian species. The morphologies of rat and mice astrocytes are pretty similar, thus we expect that FindMyCells would work in mice tissues as well. Importantly, although the rodent and the human brain share many similarities at the cellular level, there are striking differences regarding astrocytes. Human astrocytes signal faster, and they are larger and more complex^36^. Therefore, the existing DCNN may not be optimal for the detection of astrocytes in human brain sections. Nevertheless, we believe that our DCNN module can be trained further to be optimized for the detection of human astrocytes.

To promote software optimization for human applicability, we encourage the scientific community to supply their images to our team for astrocyte analysis, and thus contribute to our improving the tool. Our team is determined to provide full-scale support to FindMyCells users in the application of the software to specific tasks worldwide.

In addition to an outstanding F1 score, FindMyCells is characterized by a really short runtime. The time required for the software to detect a single cell in an image is approximately 30-fold less than the time required for a human expert for the same task. We remark here that our DCNN implementation efficiently utilizes parallel GPU capabilities. As a drawback, the annotation of the cells in the images and the training process of the DCNN module is time consuming.

Our future plans include the extension of FindMyCells with a comfortable web-based annotation and a training module, and also with a server system where users with limited GPU access can submit their annotated data set for the training of their custom DCNN. If we were supported by community efforts, we could hopefully collect versatile annotated data and develop a universal software suitable to detect various cell types.

## Electronic supplementary material

Supplementary Figure

Supplementary Dataset 1
