# Supplementary material for: A deep convolutional neural network approach for astrocyte detection
Source: Sci Rep. 2018 Aug 27;8:12878. doi: 10.1038/s41598-018-31284-x (PMC6110828; doi:10.1038/s41598-018-31284-x)
Supplement: Supplementary file 1 — Supplementary Figure [file 41598_2018_31284_MOESM1_ESM.pdf]

A deep convolutional neural network approach for astrocyte detection

Ilida Suleymanova1#, Tamas Balassa2#, Sushil Tripathi3, Csaba Molnar2, Mart Saarma1, Yulia Sidorova1, Peter Horvath2,4

1 Laboratory of Molecular Neuroscience, Research Program in Developmental Biology, Institute of Biotechnology (HiLIFE), University of Helsinki, Viikinkaari 5D, FI-00014 Helsinki, Finland.
2 Synthetic and Systems Biology Unit, Hungarian Academy of Sciences, Biological Research Centre (BRC), Temesvári körút 62, 6726 Szeged, Hungary.
3 Research Program Unit, Helsinki Institute of Life Science (HiLIFE), Faculty of Medicine, University of Helsinki, Haartmaninkatu 8, 00014 Helsinki, Finland.
4 Institute for Molecular Medicine Finland (HiLIFE), University of Helsinki, Tukholmankatu 8, 00014 Helsinki, Finland.
# I. S. and T. B. contributed equally to the research and writing of the manuscript.

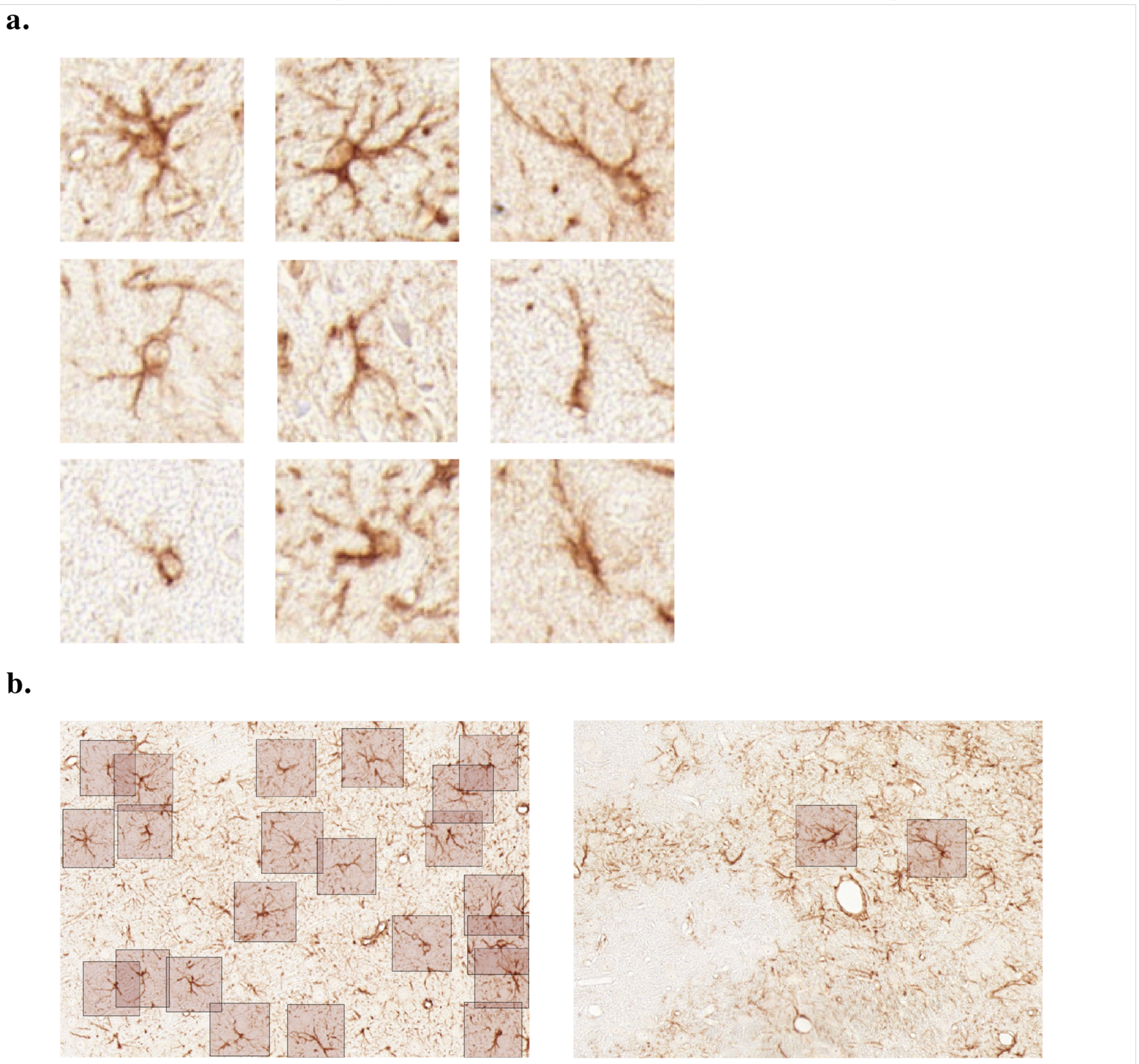

Supplemental figure 1. Examples of astrocyte in rat brain.
a: examples of the astrocyte are labeled to train DCNN.
b: detect astrocytes with the different density of astrocytes by FindMyCells.
