# Supplementary material for: A deep convolutional neural network approach for astrocyte detection
Source: Sci Rep. 2018 Aug 27;8:12878. doi: 10.1038/s41598-018-31284-x (PMC6110828; doi:10.1038/s41598-018-31284-x)
Supplement: Supplementary file 2 — Supplementary Dataset 1 [file 41598_2018_31284_MOESM2_ESM.pdf]

A deep convolutional neural network approach for astrocyte detection

Ilida Suleymanova[1#], Tamas Balassa[2#], Sushil Tripathi[3], Csaba Molnar[2],
Mart Saarma[1], Yulia Sidorova[1], Peter Horvath[2,4]

1 Laboratory of Molecular Neuroscience, Research Program in Developmental Biology,
Institute of Biotechnology (HiLIFE), University of Helsinki,
Viikinkaari 5D, FI-00014 Helsinki, Finland.
2 Synthetic and Systems Biology Unit, Hungarian Academy of Sciences,
Biological Research Centre (BRC), Temesvári körút 62, 6726 Szeged, Hungary.
3 Research Program Unit, Helsinki Institute of Life Science (HiLIFE), Faculty of Medicine,
University of Helsinki, Haartmaninkatu 8, 00014 Helsinki, Finland.
4 Institute for Molecular Medicine Finland (HiLIFE), University of Helsinki,
Tukholmankatu 8, 00014 Helsinki, Finland.
# I. S. and T. B. contributed equally to the research and writing of the manuscript.

Global

Test	Precision	Recall	Detection accuracy	F-1 score
Expert I vs Expert I Rotated images	0.89473684	0.85	0.772727273	0.8717949
Expert I Rotated images vs Expert I	0.78214286	0.82330827	0.669724771	0.8021978
Expert II vs Expert II Rotated images	0.7327044	0.79522184	0.616402116	0.7626841
Expert II Rotated images vs Expert II	0.79522184	0.73040752	0.614775726	0.7614379
Expert II vs Expert I	0.77474403	0.81071429	0.656069364	0.7923211
Expert II Rotated images vs Expert I Rotated	0.66666667	0.79699248	0.569892473	0.7260274
Expert I vs Expert II	0.81071429	0.77474403	0.656069364	0.7923211
Expert I Rotated vs Expert II Rotated	0.81203008	0.67924528	0.586956522	0.739726

Global

Test	Precision	Recall	Detection accuracy	F-1 score
Intra-Expert Accuracy (Expert I)	0.84	0.84	0.72	0.84
Intra-Expert Accuracy (Expert II)	0.76	0.76	0.62	0.76
Inter-expert Accuracy (Expert II vs Expert I)	0.77	0.77	0.62	0.76
FindMyCells	0.82	0.86	0.72	0.84
ilastik	0.23	0.59	0.20	0.33
Threshold-based method	0.05	0.11	0.04	0.07
ImageJ	0.10	0.47	0.09	0.17

Per image

Test	Precision	Recall	Detection accuracy	F-1 score
Expert I vs Expert I Rotated images	0.88280749	0.84316401	0.769345539	0.8584777
Expert I Rotated images vs Expert I	0.76800583	0.80427228	0.661254603	0.7821361
Expert II vs Expert II Rotated images	0.72999555	0.81432548	0.630762369	0.7658949
Expert II Rotated images vs Expert II	0.81432548	0.72999555	0.630762369	0.7658949
Expert II vs Expert I	0.7789183	0.80239236	0.650495524	0.7810731
Expert II Rotated images vs Expert I Rotated	0.65362636	0.78821342	0.564108227	0.7061076
Expert I vs Expert II	0.80239236	0.7789183	0.650495524	0.7810731
Expert I Rotated vs Expert II Rotated	0.80148853	0.66786934	0.580960103	0.7198395

Per image

Test	Precision	Recall	Detection accuracy	F-1 score
Intra-Expert Accuracy (Expert I)	0.83	0.82	0.72	0.82
Intra-Expert Accuracy (Expert II)	0.77	0.77	0.63	0.77
Inter-expert Accuracy (Expert II vs Expert I)	0.81	0.91	0.75	0.85
FindMyCells	0.86	0.78	0.69	0.81
ilastik	0.30	0.67	0.28	0.34
Threshold-based method	0.59	0.43	0.33	0.38
ImageJ	0.54	0.34	0.31	0.34

	Manual counting	FindMyCells	ilastik	Threshold-based method	ImageJ	Training time
**Number of cells**	280	295	715	641	1253	15000
**Time (sec)**	729	26	422	463.8158	806	115200
**Time for detecting a cell**	2.603571429	0.088135593	0.5902	0.723581591	0.64325619	7.68

Animal group	Animal number	Manual counting			FindMyCells			Spearman correlation		Pearson correlation	
		Number of cells	Average of number of cells	OIH/CTR	Number of cells	Average of number of cells	OIH/CTR	RHO	p-value	RHO	p-value
**CTR**	1	32	24.16666667	0.703448276	29	22.83333333	0.7153285	0.9489	2.5071E-06	0.9512	2.01E-06
	2	27			27						
	3	27			22						
	4	20			23						
	5	16			15						
	3	23			21						
**OIH/OIT**	7	9	17		9	16.33333333					
	8	24			22						
	9	19			19						
	10	13			11						
	11	18			18						
	12	19			19						

OIH - opioid-induced hyperalgesia

	FindMyCells									ilastik								
	**0.9**	**0.8**	**0.7**	**0.6**	**0.5**	**0.4**	**0.3**	**0.2**	**0.1**	**3500**	**2500**	**300**	**220**	**0.55**	**150**	**100**	**50**	**10**
**Recall**	0.00	0.44	0.64	0.74	0.78	0.85	0.89	0.91	1.00	0.00	0.04	0.54	0.70	0.70	0.88	0.97	1.00	1.00
**Precision**	0.88	0.88	0.99	0.94	0.85	0.83	0.76	0.68	0.59	0.64	0.64	0.36	0.29	0.25	0.21	0.14	0.07	0.07
**AUC**									0.8707									0.39

	Threshold based method									ImageJ								
	**0**	**30**	**60**	**90**	**120**	**150**	**180**	**200**	**220**	**0**	**30**	**150**	**200**	**255 1[illegible]**	**255 50 size**			
**Recall**	0.00	0.00	0.00	0.01	0.09	0.46	0.85	0.98	1.00	0.00	0.00	0.02	0.09	0.15	0.64	0.68	0.95	1.00
**Precision**	0.01	0.01	0.01	0.11	0.46	0.47	0.35	0.32	0.32	0.34	0.34	0.34	0.50	0.56	0.37	0.30	0.29	0.29
**AUC**									0.4062									0.4

AUC

FindMyCells	ilastik	Threshold-based method	ImageJ
0.8707	0.39	0.4062	0.4017

	N	Manual counting	FindMyCells							
Animal group	Animal number	Total N of cells	Total N of cells	False +	False -	True +	False +	False -	True +	Precision
CTR	**1**	32	29	3	6	26	0.09375	0.1875	0.8125	0.896551724
	**2**	27	27	7	7	20	0.259259259	0.259259259	0.74074074	0.740740741
	**3**	27	22	4	9	18	0.148148148	0.333333333	0.66666667	0.818181818
	**4**	20	23	6	3	17	0.3	0.15	0.85	0.739130435
	**5**	16	15	2	3	13	0.125	0.1875	0.8125	0.866666667
	**6**	23	21	4	6	17	0.173913043	0.260869565	0.73913043	0.80952381
OIH/OIT	**7**	9	9	1	1	8	0.111111111	0.111111111	0.88888889	0.888888889
	**8**	24	22	5	7	17	0.208333333	0.291666667	0.70833333	0.772727273
	**9**	19	19	5	5	14	0.263157895	0.263157895	0.73684211	0.736842105
	**10**	13	11	2	4	9	0.153846154	0.307692308	0.69230769	0.818181818
	**11**	18	18	4	4	14	0.222222222	0.222222222	0.77777778	0.777777778
	**12**	19	19	6	6	13	0.315789474	0.315789474	0.68421053	0.684210526
	**Average**			4.083333333	5.083333333	15.5	0.197877553	0.240841819	0.75915818	0.795785299
	**Sum**	247	235							

OIH - opioid-induced hyperalgesia

			FindMyCells			FindMyCells		
Recall	F-beta score	Detection accuracy	Striatum	Midbrain		Striatum	Midbrain	
			F-beta score	F-beta score	t-test	Detection accuracy	Detection accuracy	t-test
0.8125	0.852459016	0.742857143	0.852459016	0.833333333	0.11055341	0.742857143	0.714285714	0.08729796
0.740740741	0.740740741	0.588235294	0.740740741	0.842105263		0.588235294	0.727272727	
0.666666667	0.734693878	0.580645161	0.734693878	0.777777778		0.580645161	0.636363636	
0.85	0.790697674	0.653846154	0.790697674	0.930232558		0.653846154	0.869565217	
0.8125	0.838709677	0.722222222	0.838709677	0.866666667		0.722222222	0.764705882	
0.739130435	0.772727273	0.62962963	0.772727273	0.965517241		0.62962963	0.933333333	
0.888888889	0.888888889	0.8	0.888888889	0.909090909		0.8	0.833333333	
0.708333333	0.739130435	0.586206897	0.739130435	0.740740741		0.586206897	0.588235294	
0.736842105	0.736842105	0.583333333	0.736842105	0.833333333		0.583333333	0.714285714	
0.692307692	0.75	0.6	0.75	0.941176471		0.6	0.888888889	
0.777777778	0.777777778	0.636363636	0.777777778	0.875		0.636363636	0.777777778	
0.684210526	0.684210526	0.52	0.684210526	0.782608696		0.52	0.642857143	
0.759158181	0.775573166	0.636944956		0.742857143			0.590909091	
				0.823529412			0.7	
				0.769230769			0.625	
				0.888888889			0.8	
				0.909090909			0.833333333	
				0.615384615			0.444444444	
				0.666666667			0.5	
				0.666666667			0.5	
				0.769230769			0.625	
				0.75			0.6	
				0.769230769			0.625	
				0.888888889			0.8	
				0.742857143			0.590909091	
				0.893617021			0.807692308	
				0.815143179				

N	Images	Manual counting	Expert I vs Expert I					
			In number of cells					
		Total N of cells	Total N of cells	Absolute diffrence in N of cells	False +	False -	True +	False +
**26**	1121	12	12	0	0	0	12	0
**25**	1122	9	8	1	1	2	7	0.111111111
**24**	1123	8	6	2	0	2	6	0
**23**	1124	21	19	2	0	2	19	0
**22**	1125	16	12	4	1	5	11	0.0625
**21**	1126	15	16	1	5	4	11	0.333333333
**20**	1127	11	11	0	0	0	11	0
**19**	1128	14	12	2	2	4	10	0.142857143
**18**	1129	12	9	3	0	3	9	0
**17**	1130	17	15	2	0	2	15	0
**16**	1131	17	18	1	3	2	15	0.176470588
**15**	1132	11	11	0	1	1	10	0.090909091
**14**	1133	16	14	2	1	3	13	0.0625
**13**	1134	8	7	1	0	1	7	0
**12**	1135	6	6	0	0	0	6	0
**11**	1136	5	5	0	0	0	5	0
**10**	1137	5	4	1	0	1	4	0
**9**	1138	6	7	1	3	2	4	0.5
**8**	1139	3	3	0	1	1	2	0.333333333
**7**	1140	2	2	0	0	0	2	0
**6**	1141	5	7	2	3	1	4	0.6
**5**	1142	4	5	1	2	1	3	0.2
**4**	1143	5	5	0	1	1	4	0.2
**3**	1144	13	13	0	1	1	12	0.076923077
**2**	1145	17	17	0	2	2	15	0.117647059
**1**	1146	22	22	0	1	1	21	0.045454545
**Average**					1.076923077	1.615384615	9.153846154	0.117424588
**Sum**		280	266	26				

Rotated images

Metrics							
False -	True +	Precision	Recall	F-1 score	Detection accuracy	Total N of cells	False +
0	1	1	1	1	1	12	4
0.222222222	0.777777778	0.875	0.777777778	0.823529412	0.7	9	2
0.25	0.75	1	0.75	0.857142857	0.75	8	4
0.095238095	0.904761905	1	0.904761905	0.95	0.904761905	21	4
0.3125	0.6875	0.916666667	0.6875	0.785714286	0.647058824	16	5
0.266666667	0.733333333	0.6875	0.733333333	0.709677419	0.55	15	4
0	1	1	1	1	1	11	3
0.117647059	0.714285714	0.833333333	0.714285714	0.769230769	0.625	14	4
0.25	0.75	1	0.75	0.857142857	0.75	12	4
0.117647059	0.882352941	1	0.882352941	0.9375	0.882352941	17	2
0.117647059	0.882352941	0.833333333	0.882352941	0.857142857	0.75	17	2
0.090909091	0.909090909	0.909090909	0.909090909	0.909090909	0.833333333	11	2
0.1875	0.8125	0.928571429	0.8125	0.866666667	0.764705882	16	5
0.125	0.875	1	0.875	0.933333333	0.875	7	1
0	1	1	1	1	1	7	2
0	1	1	1	1	1	5	0
0.2	0.8	1	0.8	0.888888889	0.8	5	1
0.333333333	0.666666667	0.571428571	0.666666667	0.615384615	0.444444444	6	2
0.333333333	0.666666667	0.666666667	0.666666667	0.666666667	0.5	3	1
0	1	1	1	1	1	2	0
0.2	0.8	0.571428571	0.8	0.666666667	0.5	5	2
0.2	0.8	0.6	0.75	0.666666667	0.5	4	1
0.2	0.8	0.8	0.8	0.8	0.666666667	5	2
0.076923077	0.923076923	0.923076923	0.923076923	0.923076923	0.857142857	13	1
0.117647059	0.882352941	0.882352941	0.882352941	0.882352941	0.789473684	17	2
0.045454545	0.954545455	0.954545455	0.954545455	0.954545455	0.913043478	22	1
0.148448792	0.845087084	0.882807492	0.843164007	0.8584777	0.769345539		2.346153846
						280	

Expert I Rotated images vs Expert I

False -	True +	False +	False -	True +	Precision	Recall
4	8	0.230769231	0.307692308	0.692307692	0.666666667	0.666666667
1	7	0.428571429	0.142857143	0.857142857	0.777777778	0.875
2	4	0.666666667	0.333333333	0.666666667	0.5	0.666666667
2	17	0.15	0.1	0.9	0.80952381	0.894736842
1	11	0.416666667	0.083333333	0.916666667	0.6875	0.916666667
5	11	0.25	0.3125	0.6875	0.733333333	0.6875
3	8	0	0.3125	0.6875	0.727272727	0.727272727
2	10	0.333333333	0.166666667	0.833333333	0.714285714	0.833333333
1	8	0.625	0.125	0.875	0.666666667	0.888888889
0	15	0.133333333	0	1	0.882352941	1
3	15	0.111111111	0.166666667	0.833333333	0.882352941	0.833333333
2	9	0.3	0.2	0.8	0.818181818	0.818181818
3	11	0.266666667	0.2	0.8	0.6875	0.785714286
1	6	0.125	0.125	0.875	0.857142857	0.857142857
1	5	0.166666667	0.166666667	0.833333333	0.714285714	0.833333333
0	5	0	0	1	1	1
0	4	0.25	0	1	0.8	1
3	4	0.285714286	0.428571429	0.571428571	0.666666667	0.571428571
1	2	0.333333333	0.333333333	0.666666667	0.666666667	0.666666667
0	2	1	0	1	1	1
4	3	0.6	0.6	0.4	0.6	0.428571429
2	3	0.666666667	0.333333333	0.666666667	0.75	0.6
2	3	0.75	0.5	0.5	0.6	0.6
1	12	0.076923077	0.076923077	0.923076923	0.923076923	0.923076923
2	15	0.117647059	0.117647059	0.882352941	0.882352941	0.882352941
1	21	0.045454545	0.045454545	0.954545455	0.954545455	0.954545455
1.807692308	8.423076923	0.32036631	0.199133804	0.800866196	0.768005832	0.804272285

				In number of cells		
F-1 score	Detection accuracy	Total N of cells	Absolute diffrence in N of cells	False +	False -	True +
0.666666667	0.5	13	1	2	1	11
0.823529412	0.7	8	1	0	1	8
0.571428571	0.4	6	2	1	3	5
0.85	0.739130435	20	1	4	5	16
0.785714286	0.647058824	12	4	2	6	10
0.709677419	0.55	16	1	3	2	13
0.727272727	0.571428571	13	2	3	1	10
0.769230769	0.625	21	7	8	1	13
0.761904762	0.615384615	16	4	6	2	10
0.9375	0.882352941	18	1	6	5	12
0.857142857	0.75	19	2	5	3	14
0.818181818	0.692307692	13	2	3	1	10
0.733333333	0.578947368	13	3	1	4	12
0.857142857	0.75	8	0	1	1	7
0.769230769	0.625	5	1	0	1	5
1	1	3	2	0	2	3
0.888888889	0.8	5	0	1	1	4
0.615384615	0.444444444	9	3	4	1	5
0.666666667	0.5	3	0	1	1	2
1	1	3	1	1	0	2
0.5	0.333333333	5	0	1	1	4
0.666666667	0.5	4	0	2	2	2
0.6	0.428571429	7	2	3	1	4
0.923076923	0.857142857	14	1	2	1	12
0.882352941	0.789473684	15	2	1	3	14
0.954545455	0.913043478	24	2	5	3	19
0.782136092	0.661254603			2.538461538	2.038461538	8.730769231
		293	45			

Expert II vs Expert I

Metrics						
**False +**	**False -**	**True +**	**Precision**	**Recall**	**F-1 score**	**Detection accuracy**
0.153846154	0.083333333	0.916666667	0.846153846	0.916666667	0.88	0.785714286
0	0.111111111	0.888888889	1	0.888888889	0.941176471	0.888888889
0.166666667	0.375	0.625	0.833333333	0.625	0.714285714	0.555555556
0.2	0.238095238	0.761904762	0.8	0.761904762	0.780487805	0.64
0.166666667	0.375	0.625	0.833333333	0.625	0.714285714	0.555555556
0.1875	0.133333333	0.866666667	0.8125	0.866666667	0.838709677	0.722222222
0.230769231	0.090909091	0.909090909	0.769230769	0.909090909	0.833333333	0.714285714
0.380952381	0.071428571	0.928571429	0.619047619	0.928571429	0.742857143	0.590909091
0.375	0.166666667	0.833333333	0.625	0.833333333	0.714285714	0.555555556
0.333333333	0.294117647	0.705882353	0.666666667	0.705882353	0.685714286	0.52173913
0.263157895	0.176470588	0.823529412	0.736842105	0.823529412	0.777777778	0.636363636
0.230769231	0.090909091	0.909090909	0.769230769	0.909090909	0.833333333	0.714285714
0.076923077	0.25	0.75	0.923076923	0.75	0.827586207	0.705882353
0.125	0.125	0.875	0.875	0.875	0.875	0.777777778
0	0.166666667	0.833333333	1	0.833333333	0.909090909	0.833333333
0	0.4	0.6	1	0.6	0.75	0.6
0.2	0.2	0.8	0.8	0.8	0.8	0.666666667
0.444444444	0.166666667	0.833333333	0.555555556	0.833333333	0.666666667	0.5
0.333333333	0.333333333	0.666666667	0.666666667	0.666666667	0.666666667	0.5
0.333333333	0	1	0.666666667	1	0.8	0.666666667
0.2	0.2	0.8	0.8	0.8	0.8	0.666666667
0.5	0.5	0.5	0.5	0.5	0.5	0.333333333
0.428571429	0.2	0.8	0.571428571	0.8	0.666666667	0.5
0.142857143	0.076923077	0.923076923	0.857142857	0.923076923	0.888888889	0.8
0.066666667	0.176470588	0.823529412	0.933333333	0.823529412	0.875	0.777777778
0.208333333	0.136363636	0.863636364	0.791666667	0.863636364	0.826086957	0.703703704
0.221081705	0.19760764	0.80239236	0.778918295	0.80239236	0.781073074	0.650495524

					Expert I vs Expert II
In number of cells			Metrics		
False +	False -	True +	False +	False -	True +
1	2	11	0.076923077	0.153846154	0.846153846
1	0	8	0.125	0	1
3	1	5	0.5	0.166666667	0.833333333
5	4	16	0.25	0.2	0.8
6	2	10	0.5	0.166666667	0.833333333
2	3	13	0.125	0.1875	0.8125
1	3	10	0.076923077	0.230769231	0.769230769
1	8	13	0.047619048	0.380952381	0.619047619
2	6	10	0.125	0.375	0.625
5	6	12	0.277777778	0.333333333	0.666666667
3	5	14	0.157894737	0.263157895	0.736842105
1	3	10	0.076923077	0.230769231	0.769230769
4	1	12	0.307692308	0.076923077	0.923076923
1	1	7	0.125	0.125	0.875
1	0	5	0.2	0	1
2	0	3	0.666666667	0	1
1	1	4	0.2	0.2	0.8
1	4	5	0.111111111	0.444444444	0.555555556
1	1	2	0.333333333	0.333333333	0.666666667
0	1	2	0	0.333333333	0.666666667
1	1	4	0.2	0.2	0.8
2	2	2	0.5	0.5	0.5
1	3	4	0.142857143	0.428571429	0.571428571
1	2	12	0.071428571	0.142857143	0.857142857
3	1	14	0.2	0.066666667	0.933333333
3	5	19	0.125	0.208333333	0.791666667
2.038461538	2.538461538	8.730769231	0.212390382	0.221081705	0.778918295

Precision	Recall	F-1 score	Detection accuracy	Total N of cells	False +
0.916666667	0.846153846	0.88	0.785714286	13	3
0.888888889	1	0.941176471	0.888888889	9	2
0.625	0.833333333	0.714285714	0.555555556	6	1
0.761904762	0.8	0.780487805	0.64	25	8
0.625	0.833333333	0.714285714	0.555555556	12	1
0.866666667	0.8125	0.838709677	0.722222222	16	6
0.909090909	0.769230769	0.833333333	0.714285714	12	2
0.928571429	0.619047619	0.742857143	0.590909091	20	2
0.833333333	0.625	0.714285714	0.555555556	16	3
0.705882353	0.666666667	0.685714286	0.52173913	19	6
0.823529412	0.736842105	0.777777778	0.636363636	21	5
0.909090909	0.769230769	0.833333333	0.714285714	12	3
0.75	0.923076923	0.827586207	0.705882353	14	4
0.875	0.875	0.875	0.777777778	8	3
0.833333333	1	0.909090909	0.833333333	5	1
0.6	1	0.75	0.6	4	1
0.8	0.8	0.8	0.666666667	5	1
0.833333333	0.555555556	0.666666667	0.5	11	4
0.666666667	0.666666667	0.666666667	0.5	4	1
1	0.666666667	0.8	0.666666667	4	1
0.8	0.8	0.8	0.666666667	8	3
0.5	0.5	0.5	0.333333333	6	4
0.8	0.571428571	0.666666667	0.5	9	2
0.923076923	0.857142857	0.888888889	0.8	16	5
0.823529412	0.933333333	0.875	0.777777778	18	6
0.863636364	0.791666667	0.826086957	0.703703704	25	7
0.80239236	0.778918295	0.781073074	0.650495524		3.269230769
				318	

Expert II vs Expert II Rotated images

In number of cells		Metrics			
False -	True +	False +	False -	True +	Precision
3	10	0.230769231	0.230769231	0.769230769	0.769230769
1	7	0.222222222	0.125	0.875	0.777777778
1	5	0.166666667	0.166666667	0.833333333	0.833333333
3	17	0.32	0.15	0.85	0.68
1	11	0.083333333	0.083333333	0.916666667	0.916666667
6	10	0.375	0.375	0.625	0.625
3	10	0.166666667	0.230769231	0.769230769	0.833333333
3	18	0.1	0.142857143	0.857142857	0.9
3	13	0.1875	0.1875	0.8125	0.8125
5	13	0.315789474	0.277777778	0.722222222	0.684210526
3	16	0.238095238	0.157894737	0.842105263	0.761904762
4	9	0.25	0.307692308	0.692307692	0.75
3	10	0.285714286	0.230769231	0.769230769	0.714285714
3	5	0.375	0.375	0.625	0.625
1	4	0.2	0.2	0.8	0.8
0	3	0.25	0	1	0.75
1	4	0.2	0.2	0.8	0.8
2	7	0.363636364	0.222222222	0.777777778	0.636363636
0	3	0.25	0	1	0.75
0	3	0.25	0	1	0.75
0	5	0.375	0	1	0.625
2	2	0.666666667	0.5	0.5	0.333333333
0	7	0.222222222	0	1	0.777777778
3	11	0.3125	0.214285714	0.785714286	0.6875
3	12	0.333333333	0.2	0.8	0.666666667
6	18	0.28	0.25	0.75	0.72
2.307692308	8.961538462	0.27000445	0.185674523	0.814325477	0.72999555

Recall	F-1 score	Detection accuracy	Total N of cells	False +	False -
0.769230769	0.769230769	0.625	13	3	3
0.875	0.823529412	0.7	8	1	2
0.833333333	0.833333333	0.714285714	6	1	1
0.85	0.755555556	0.607142857	20	3	8
0.916666667	0.916666667	0.846153846	12	1	1
0.625	0.625	0.454545455	16	6	6
0.769230769	0.8	0.666666667	13	3	2
0.857142857	0.87804878	0.782608696	21	3	2
0.8125	0.8125	0.684210526	16	3	3
0.722222222	0.702702703	0.541666667	18	5	6
0.842105263	0.8	0.666666667	19	3	5
0.692307692	0.72	0.5625	13	4	3
0.769230769	0.740740741	0.588235294	13	3	4
0.625	0.625	0.454545455	8	3	3
0.8	0.8	0.666666667	5	1	1
1	0.857142857	0.75	3	0	1
0.8	0.8	0.666666667	5	1	1
0.777777778	0.7	0.538461538	9	2	4
1	0.857142857	0.75	3	0	1
1	0.857142857	0.75	3	0	1
1	0.769230769	0.625	5	0	3
0.5	0.4	0.25	4	2	4
1	0.875	0.777777778	7	0	2
0.785714286	0.733333333	0.578947368	14	3	5
0.8	0.727272727	0.571428571	15	3	6
0.75	0.734693878	0.580645161	24	6	7
0.814325477	0.765894894	0.630762369		2.307692308	3.269230769
			293		

Expert II Rotated images vs Expert II

True +	False +	False -	True +	Precision	Recall
10	0.230769231	0.230769231	0.769230769	0.769230769	0.769230769
7	0.111111111	0.222222222	0.777777778	0.875	0.777777778
5	0.166666667	0.166666667	0.833333333	0.833333333	0.833333333
17	0.12	0.32	0.68	0.85	0.68
11	0.083333333	0.083333333	0.916666667	0.916666667	0.916666667
10	0.375	0.375	0.625	0.625	0.625
10	0.25	0.166666667	0.833333333	0.769230769	0.833333333
18	0.15	0.1	0.9	0.857142857	0.9
13	0.1875	0.1875	0.8125	0.8125	0.8125
13	0.263157895	0.315789474	0.684210526	0.722222222	0.684210526
16	0.095238095	0.238095238	0.761904762	0.842105263	0.761904762
9	0.333333333	0.25	0.75	0.692307692	0.75
10	0.214285714	0.285714286	0.714285714	0.769230769	0.714285714
5	0.375	0.375	0.625	0.625	0.625
4	0.2	0.2	0.8	0.8	0.8
3	0	0.25	0.75	1	0.75
4	0.2	0.2	0.8	0.8	0.8
7	0.181818182	0.363636364	0.636363636	0.777777778	0.636363636
3	0	0.25	0.75	1	0.75
3	0	0.25	0.75	1	0.75
5	0	0.375	0.625	1	0.625
2	0.333333333	0.666666667	0.333333333	0.5	0.333333333
7	0	0.222222222	0.777777778	1	0.777777778
11	0.1875	0.3125	0.6875	0.785714286	0.6875
12	0.166666667	0.333333333	0.666666667	0.8	0.666666667
18	0.2	0.28	0.72	0.75	0.72
8.961538462	0.170181291	0.27000445	0.72999555	0.814325477	0.72999555

F-1 score	Detection accuracy	Total N of cells	False +	False -	True +
0.769230769	0.625	13	3	2	10
0.823529412	0.7	9	2	1	7
0.833333333	0.714285714	6	2	2	4
0.755555556	0.607142857	25	7	1	18
0.916666667	0.846153846	12	4	4	8
0.625	0.454545455	16	5	5	11
0.8	0.666666667	12	6	5	6
0.87804878	0.782608696	20	8	0	12
0.8125	0.684210526	16	7	0	9
0.702702703	0.541666667	19	4	0	15
0.8	0.666666667	21	5	2	16
0.72	0.5625	12	2	1	10
0.740740741	0.588235294	14	6	6	8
0.625	0.454545455	8	4	3	4
0.8	0.666666667	5	2	3	3
0.857142857	0.75	4	0	1	4
0.8	0.666666667	5	1	0	4
0.7	0.538461538	11	7	3	4
0.857142857	0.75	4	1	0	3
0.857142857	0.75	4	2	0	2
0.769230769	0.625	8	4	3	4
0.4	0.25	6	4	3	2
0.875	0.777777778	9	4	0	5
0.733333333	0.578947368	16	5	2	11
0.727272727	0.571428571	18	4	3	14
0.734693878	0.580645161	25	7	4	18
0.765894894	0.630762369		4.076923077	2.076923077	8.153846154
		318			

Expert II Rotated images vs Expert I Rotated

False +	False -	True +	Precision	Recall	F-1 score
0.153846154	0.153846154	0.846153846	0.769230769	0.833333333	0.8
0.428571429	0.142857143	0.857142857	0.777777778	0.875	0.823529412
0.333333333	0.333333333	0.666666667	0.666666667	0.666666667	0.666666667
0.3	0.05	0.95	0.72	0.947368421	0.818181818
0.333333333	0.333333333	0.666666667	0.666666667	0.666666667	0.666666667
0.3125	0.3125	0.6875	0.6875	0.6875	0.6875
0.1875	0.4375	0.5625	0.5	0.545454545	0.52173913
0.666666667	0	1	0.6	1	0.75
0.875	0	1	0.5625	1	0.72
0.266666667	0	1	0.789473684	1	0.882352941
0.277777778	0.111111111	0.888888889	0.761904762	0.888888889	0.820512821
0.3	0.1	0.9	0.833333333	0.909090909	0.869565217
0.333333333	0.4	0.6	0.571428571	0.571428571	0.571428571
0.125	0.375	0.625	0.5	0.571428571	0.533333333
0.333333333	0.5	0.5	0.6	0.5	0.545454545
0	0.2	0.8	1	0.8	0.888888889
0.25	0	1	0.8	1	0.888888889
1	0.428571429	0.571428571	0.363636364	0.571428571	0.444444444
0.333333333	0	1	0.75	1	0.857142857
3	0	1	0.5	1	0.666666667
1	0.4	0.6	0.5	0.571428571	0.533333333
1.666666667	0.666666667	0.333333333	0.333333333	0.4	0.363636364
1.25	0	1	0.555555556	1	0.714285714
0.384615385	0.153846154	0.846153846	0.6875	0.846153846	0.75862069
0.235294118	0.176470588	0.823529412	0.777777778	0.823529412	0.8
0.272727273	0.181818182	0.818181818	0.72	0.818181818	0.765957447
0.562288415	0.209879004	0.790120996	0.653626356	0.788213415	0.706107554

	Expert I Rotated vs				
**Detection accuracy**	**False +**	**False -**	**True +**	**False +**	**False -**
0.666666667	2	3	10	0.153846154	0.230769231
0.7	1	2	7	0.111111111	0.222222222
0.5	3	3	3	0.5	0.5
0.692307692	2	8	17	0.08	0.32
0.5	4	4	8	0.333333333	0.333333333
0.523809524	5	5	11	0.3125	0.3125
0.352941176	2	3	9	0.166666667	0.25
0.6	0	8	12	0	0.4
0.5625	0	7	9	0	0.4375
0.789473684	0	4	15	0.210526316	0.210526316
0.695652174	2	5	16	0.095238095	0.238095238
0.769230769	2	3	9	0.166666667	0.25
0.4	5	5	9	0.357142857	0.357142857
0.363636364	2	3	5	0.25	0.375
0.375	2	1	4	0.4	0.2
0.8	1	0	4	0.25	0
0.8	0	1	4	0	0.2
0.285714286	3	7	4	0.272727273	0.636363636
0.75	0	1	3	0	0.25
0.5	0	2	2	0	0.5
0.363636364	2	3	5	0.25	0.375
0.222222222	4	5	1	0.666666667	0.833333333
0.555555556	0	4	5	0	0.444444444
0.611111111	2	5	11	0.125	0.3125
0.666666667	2	3	15	0.111111111	0.166666667
0.620689655	4	7	18	0.16	0.28
0.564108227	1.923076923	3.923076923	8.307692308	0.191251394	0.332130665

s Expert II Rotated

True +	Precision	Recall	F-1 score	Detection accuracy	Total N of cells	Absolute diffrence in N of cells
0.769230769	0.833333333	0.769230769	0.8	0.666666667	12	0
0.777777778	0.875	0.777777778	0.823529412	0.7	10	1
0.5	0.5	0.5	0.5	0.333333333	10	2
0.68	0.894736842	0.68	0.772727273	0.62962963	22	1
0.666666667	0.666666667	0.666666667	0.666666667	0.5	14	2
0.6875	0.6875	0.6875	0.6875	0.523809524	14	1
0.75	0.818181818	0.75	0.782608696	0.642857143	11	0
0.6	1	0.6	0.75	0.6	13	1
0.5625	1	0.5625	0.72	0.5625	12	0
0.789473684	1	0.789473684	0.882352941	0.789473684	17	0
0.761904762	0.888888889	0.761904762	0.820512821	0.695652174	15	2
0.75	0.818181818	0.75	0.782608696	0.642857143	12	1
0.642857143	0.642857143	0.642857143	0.642857143	0.473684211	19	3
0.625	0.714285714	0.625	0.666666667	0.5	9	1
0.8	0.666666667	0.8	0.727272727	0.571428571	7	1
1	0.8	1	0.888888889	0.8	4	1
0.8	1	0.8	0.888888889	0.8	6	1
0.363636364	0.571428571	0.363636364	0.444444444	0.285714286	7	1
0.75	1	0.75	0.857142857	0.75	3	0
0.5	1	0.5	0.666666667	0.5	1	1
0.625	0.714285714	0.625	0.666666667	0.5	8	3
0.166666667	0.2	0.166666667	0.181818182	0.1	4	0
0.555555556	1	0.555555556	0.714285714	0.555555556	8	3
0.6875	0.846153846	0.6875	0.75862069	0.611111111	14	1
0.833333333	0.882352941	0.833333333	0.857142857	0.75	18	1
0.72	0.818181818	0.72	0.765957447	0.620689655	25	3
0.667869335	0.80148853	0.667869335	0.719839475	0.580960103		
					295	31

FindMyCells

In number of cells			Metrics					
**False +**	**False -**	**True +**	**False +**	**False -**	**True +**	**Precision**	**Recall**	**F-1 score**
2	2	10	0.1667	0.166666667	0.833333333	0.833333333	0.833333333	0.833333333
2	1	8	0.2222	0.111111111	0.888888889	0.8	0.888888889	0.842105263
3	1	7	0.375	0.125	0.875	0.7	0.875	0.777777778
2	1	20	0.0952	0.047619048	0.952380952	0.909090909	0.952380952	0.930232558
1	3	13	0.0625	0.1875	0.8125	0.928571429	0.8125	0.866666667
0	1	14	0	0.066666667	0.933333333	1	0.933333333	0.965517241
1	1	10	0.0909	0.090909091	0.909090909	0.909090909	0.909090909	0.909090909
3	4	10	0.2143	0.285714286	0.714285714	0.769230769	0.714285714	0.740740741
2	2	10	0.1667	0.166666667	0.833333333	0.833333333	0.833333333	0.833333333
1	1	16	0.0588	0.058823529	0.941176471	0.941176471	0.941176471	0.941176471
1	3	14	0.0588	0.176470588	0.823529412	0.933333333	0.823529412	0.875
3	2	9	0.2727	0.181818182	0.818181818	0.75	0.818181818	0.782608696
6	3	13	0.375	0.1875	0.8125	0.684210526	0.8125	0.742857143
2	1	7	0.25	0.125	0.875	0.777777778	0.875	0.823529412
2	1	5	0.3333	0.166666667	0.833333333	0.714285714	0.833333333	0.769230769
0	1	4	0	0.2	0.8	1	0.8	0.888888889
1	0	5	0.2	0	1	0.833333333	1	0.909090909
3	2	4	0.5	0.333333333	0.666666667	0.571428571	0.666666667	0.615384615
1	1	2	0.3333	0.333333333	0.666666667	0.666666667	0.666666667	0.666666667
0	1	1	0	0.5	0.5	1	0.5	0.666666667
3	0	5	0.6	0	1	0.625	1	0.769230769
1	1	3	0.25	0.25	0.75	0.75	0.75	0.75
3	0	5	0.6	0	1	0.625	1	0.769230769
2	1	12	0.1538	0.076923077	0.923076923	0.857142857	0.923076923	0.888888889
5	4	13	0.2941	0.235294118	0.764705882	0.722222222	0.764705882	0.742857143
4	1	21	0.1818	0.045454545	0.954545455	0.84	0.954545455	0.893617021
2.076923077	1.5	9.269230769	0.2252	0.158402727	0.841597273	0.806701083	0.841597273	0.815143179
		295						

	ilastik						
	In number of cells					Metrics	
Detection accuracy	Total N of cells	Absolute diffrence in N of cells	False +	False -	True +	False +	False -
0.714285714	23	11	13	2	10	1.083333333	0.166666667
0.727272727	18	9	12	3	6	1.333333333	0.333333333
0.636363636	61	53	57	4	4	7.125	0.5
0.869565217	64	43	55	12	9	2.619047619	0.571428571
0.764705882	13	3	5	8	8	0.3125	0.5
0.933333333	31	16	22	6	9	1.466666667	0.4
0.833333333	1	10	1	11	0	0.090909091	1
0.588235294	2	12	1	13	1	0.071428571	0.928571429
0.714285714	2	10	2	12	0	0.166666667	1
0.888888889	18	1	5	4	13	0.294117647	0.235294118
0.777777778	49	32	33	1	16	1.941176471	0.058823529
0.642857143	14	3	8	5	6	0.727272727	0.454545455
0.590909091	56	40	43	3	13	2.6875	0.1875
0.7	45	37	39	2	6	4.875	0.25
0.625	43	37	39	2	4	6.5	0.333333333
0.8	12	7	8	1	4	1.6	0.2
0.833333333	7	2	3	1	4	0.6	0.2
0.444444444	23	17	18	1	5	3	0.166666667
0.5	7	4	4	0	3	1.333333333	0
0.5	4	2	3	1	1	1.5	0.5
0.625	42	37	38	1	4	7.6	0.2
0.6	64	60	60	0	4	15	0
0.625	52	47	50	3	2	10	0.6
0.8	22	9	13	4	9	1	0.307692308
0.590909091	31	14	17	3	14	1	0.176470588
0.807692308	11	11	1	12	10	0.045454545	0.545454545
0.697430497			21.153846	4.4230769	6.346153846	2.845105385	0.377530021
	715	527					

True +	Precision	Recall	F-1 score	Detection accuracy	Total N of cells	Absolute diffrence in N of cells
0.833333333	0.434782609	0.833333333	0.571428571	0.4	31	19
0.666666667	0.333333333	0.666666667	0.444444444	0.285714286	32	23
0.5	0.06557377	0.5	0.115942029	0.061538462	43	35
0.428571429	0.140625	0.428571429	0.211764706	0.118421053	44	23
0.5	0.615384615	0.5	0.551724138	0.380952381	3	13
0.6	0.290322581	0.6	0.391304348	0.243243243	8	7
0	0	0	0	0	28	17
0.071428571	0.5	0.071428571	0.125	0.066666667	3	11
0	0	0	0	0	1	11
0.764705882	0.722222222	0.764705882	0.742857143	0.590909091	28	11
0.941176471	0.326530612	0.941176471	0.484848485	0.32	28	11
0.545454545	0.428571429	0.545454545	0.48	0.315789474	12	1
0.8125	0.232142857	0.8125	0.361111111	0.220338983	25	9
0.75	0.133333333	0.75	0.226415094	0.127659574	35	27
0.666666667	0.093023256	0.666666667	0.163265306	0.088888889	59	53
0.8	0.333333333	0.8	0.470588235	0.307692308	8	3
0.8	0.571428571	0.8	0.666666667	0.5	26	21
0.833333333	0.217391304	0.833333333	0.344827586	0.208333333	51	45
1	0.428571429	1	0.6	0.428571429	27	24
0.5	0.25	0.5	0.333333333	0.2	10	8
0.8	0.095238095	0.8	0.170212766	0.093023256	26	21
1	0.0625	1	0.117647059	0.0625	61	57
0.4	0.038461538	0.4	0.070175439	0.036363636	24	19
0.692307692	0.409090909	0.692307692	0.514285714	0.346153846	15	2
0.823529412	0.451612903	0.823529412	0.583333333	0.411764706	9	8
0.454545455	0.909090909	0.454545455	0.606060606	0.434782609	4	18
0.622469979	0.31086787	0.622469979	0.359509081	0.24035797		
					641	497

Threshold based method

In number of cells			Metrics					
False +	False -	True +	False +	False -	True +	Precision	Recall	F-1 score
31	12	0	0	1	0	0	0	0
32	9	0	0	1	0	0	0	0
43	8	0	0	1	0	0	0	0
44	21	0	0	1	0	0	0	0
0	13	3	0	0.785714286	0.214285714	1	0.1875	0.315789474
4	11	4	0.266666667	0.733333333	0.266666667	0.5	0.26666667	0.347826087
28	11	0	0	1	0	0	0	0
1	12	2	0	0.833333333	0.166666667	0.66666667	0.14285714	0.235294118
0	11	1	0	0.888888889	0.111111111	1	0.08333333	0.153846154
16	5	12	1.461538462	0.307692308	0.692307692	0.42857143	0.70588235	0.533333333
28	17	0	0	1	0	0	0	0
12	11	0	0	1	0	0	0	0
25	16	0	0	1	0	0	0	0
35	8	0	0	1	0	0	0	0
59	6	0	0	1	0	0	0	0
4	1	4	0.8	0.2	0.8	0.5	0.8	0.615384615
23	2	3	4.6	0.4	0.6	0.11538462	0.6	0.193548387
51	6	0	0	1	0	0	0	0
27	3	0	0	1	0	0	0	0
10	2	0	0	1	0	0	0	0
26	5	0	0	1	0	0	0	0
61	4	0	0	1	0	0	0	0
24	5	0	0	1	0	0	0	0
15	13	0	0	1	0	0	0	0
9	17	0	0	1	0	0	0	0
1	19	3	0.047619048	0.857142857	0.142857143	0.75	0.13636364	0.230769231
23.42307692	9.538461538	1.230769231	0.275993238	0.884850193	0.115149807	0.19079318	0.11240781	0.100991977

	ImageJ							
			In number of cells			Metrics		
Detection accuracy	Total N of cells	Absolute diffrence in N of cells	False +	False -	True +	False +	False -	True +
0	58	46	58	12	0	4.83333333	1	0
0	65	53	65	9	0	7.22222222	1	0
0	91	82	89	7	2	11.125	0.75	0.25
0	74	66	72	6	2	3.42857143	0.9047619	0.0952381
0.1875	12	9	1	10	11	0.0625	0.3125	0.6875
0.210526316	29	13	18	5	11	1.2	0.26666667	0.73333333
0	69	54	65	11	4	5.90909091	0.63636364	0.36363636
0.133333333	25	14	15	1	10	1.07142857	0.28571429	0.71428571
0.083333333	8	4	1	5	7	0.08333333	0.41666667	0.58333333
0.363636364	58	41	49	8	9	2.88235294	0.47058824	0.52941176
0	41	24	33	9	8	1.94117647	0.52941176	0.47058824
0	20	9	14	5	6	1.27272727	0.45454545	0.54545455
0	36	20	25	5	11	1.5625	0.3125	0.6875
0	66	58	62	4	4	7.75	0.5	0.5
0	97	91	96	5	1	16	0.83333333	0.16666667
0.444444444	17	12	15	3	2	3	0.6	0.4
0.107142857	61	56	59	3	2	11.8	0.6	0.4
0	73	67	73	6	0	12.1666667	1	0
0	60	57	60	3	0	20	1	0
0	24	22	23	1	1	11.5	0.5	0.5
0	53	48	50	2	3	10	0.4	0.6
0	97	93	97	4	0	24.25	1	0
0	53	48	51	3	2	10.2	0.6	0.4
0	20	7	12	5	8	0.92307692	0.38461538	0.61538462
0	29	12	16	4	13	0.94117647	0.23529412	0.76470588
0.130434783	17	5	4	9	13	0.18181818	0.40909091	0.59090909
0.06385967			43.1923077	5.57692308	5	6.5887298	0.59238663	0.40761337
	1253	1011						

Precision	Recall	F-1 score	Detection accuracy
0	0	0	0
0	0	0	0
0.02197802	0.22222222	0.04	0.020408163
0.02702703	0.25	0.048780488	0.025
0.91666667	0.52380952	0.666666667	0.5
0.37931034	0.6875	0.488888889	0.323529412
0.05797101	0.26666667	0.095238095	0.05
0.4	0.90909091	0.555555556	0.384615385
0.875	0.58333333	0.7	0.538461538
0.15517241	0.52941176	0.24	0.136363636
0.19512195	0.47058824	0.275862069	0.16
0.3	0.54545455	0.387096774	0.24
0.30555556	0.6875	0.423076923	0.268292683
0.06060606	0.5	0.108108108	0.057142857
0.01030928	0.16666667	0.019417476	0.009803922
0.11764706	0.4	0.181818182	0.1
0.03278689	0.4	0.060606061	0.03125
0	0	0	0
0	0	0	0
0.04166667	0.5	0.076923077	0.04
0.05660377	0.6	0.103448276	0.054545455
0	0	0	0
0.03773585	0.4	0.068965517	0.035714286
0.4	0.61538462	0.484848485	0.32
0.44827586	0.76470588	0.565217391	0.393939394
0.76470588	0.59090909	0.666666667	0.5
0.21554386	0.40820167	0.24066095	0.161117951

N	Manual counting	Expert I vs Expert II								
		In number of cells					Metrics			
	Total N of cells	Total N of cells	Absolute diffrence in N of cells	False +	False -	True +	False +	False -	True +	Precision
**1**	14	18	4	4	0	14	0.22222222	0	1	0.77777778
**2**	8	14	6	6	0	8	0.42857143	0	1	0.57142857
**3**	14	17	3	3	0	14	0.17647059	0	1	0.82352941
**4**	16	17	1	1	0	16	0.05882353	0	1	0.94117647
**5**	24	28	4	5	1	23	0.17857143	0.04166667	0.95833333	0.82142857
**6**	14	16	2	2	0	14	0.125	0	1	0.875
**7**	21	26	5	5	0	21	0.19230769	0	1	0.80769231
**8**	8	10	2	2	0	8	0.2	0	1	0.8
**9**	16	18	2	2	0	16	0.11111111	0	1	0.88888889
**10**	8	11	3	3	0	8	0.27272727	0	1	0.72727273
**11**	15	16	1	1	0	15	0.0625	0	1	0.9375
**12**	15	14	1	1	2	13	0.07142857	0.13333333	0.86666667	0.92857143
**13**	12	17	5	5	0	12	0.29411765	0	1	0.70588235
**14**	14	20	6	7	1	13	0.35	0.07142857	0.92857143	0.65
**15**	4	12	8	8	0	4	0.66666667	0	1	0.33333333
**16**	15	21	6	6	0	15	0.28571429	0	1	0.71428571
**17**	8	13	5	5	0	8	0.38461538	0	1	0.61538462
**18**	9	18	9	9	0	9	0.5	0	1	0.5
**19**	8	12	4	4	0	8	0.33333333	0	1	0.66666667
**20**	10	13	3	3	0	10	0.23076923	0	1	0.76923077
**21**	6	14	8	8	0	6	0.57142857	0	1	0.42857143
**22**	9	12	3	3	0	9	0.25	0	1	0.75
**23**	15	18	3	3	0	15	0.16666667	0	1	0.83333333
**24**	17	19	2	2	0	17	0.10526316	0	1	0.89473684
**25**	16	23	7	7	0	16	0.30434783	0	1	0.69565217
**26**	7	20	13	13	0	7	0.65	0	1	0.35

**27**	7	10	3	3	0	7	0.3	0	1	0.7
**28**	18	20	2	3	1	17	0.15	0.05555556	0.94444444	0.85
**29**	13	15	2	2	0	13	0.13333333	0	1	0.86666667
**30**	19	21	2	2	0	19	0.0952381	0	1	0.9047619
**31**	9	9	0	0	0	9	0	0	1	1
**32**	14	14	0	0	0	14	0	0	1	1
**33**	21	22	1	2	1	20	0.09090909	0.04761905	0.95238095	0.90909091
**34**	18	22	4	4	0	18	0.18181818	0	1	0.81818182
**35**	26	30	4	5	1	25	0.16666667	0.03846154	0.96153846	0.83333333
**36**	23	25	2	3	1	22	0.12	0.04347826	0.95652174	0.88
**37**	20	21	1	3	2	18	0.14285714	0.1	0.9	0.85714286
**38**	20	22	2	3	1	19	0.13636364	0.05	0.95	0.86363636
**39**	21	22	1	4	3	18	0.18181818	0.14285714	0.85714286	0.81818182
**40**	26	29	3	3	0	26	0.10344828	0	1	0.89655172
**41**	19	19	0	2	2	17	0.10526316	0.10526316	0.89473684	0.89473684
**42**	18	18	0	1	1	17	0.05555556	0.05555556	0.94444444	0.94444444
**43**	21	23	2	2	0	21	0.08695652	0	1	0.91304348
**44**	14	14	0	1	1	13	0.07142857	0.07142857	0.92857143	0.92857143
**45**	12	12	0	4	4	8	0.33333333	0.33333333	0.66666667	0.66666667
**46**	22	21	1	1	2	20	0.04761905	0.09090909	0.90909091	0.95238095
**47**	3	4	1	1	0	3	0.25	0	1	0.75
**48**	17	17	0	4	4	13	0.23529412	0.23529412	0.76470588	0.76470588
**49**	18	19	1	2	1	17	0.10526316	0.05555556	0.94444444	0.89473684
**50**	14	16	2	3	1	13	0.1875	0.07142857	0.92857143	0.8125
**51**	14	16	2	3	1	13	0.1875	0.07142857	0.92857143	0.8125
**52**	10	10	0	0	0	10	0	0	1	1
**53**	11	14	3	4	1	10	0.28571429	0.09090909	0.90909091	0.71428571
**54**	12	12	0	2	2	10	0.16666667	0.16666667	0.83333333	0.83333333
**55**	29	32	3	3	0	29	0.09375	0	1	0.90625
**56**	24	25	1	2	1	23	0.08	0.04166667	0.95833333	0.92
**57**	29	37	8	8	0	29	0.21621622	0	1	0.78378378
**58**	18	20	2	2	0	18	0.1	0	1	0.9

**59**	17	17	0	1	1	16	0.05882353	0.05882353	0.94117647	0.94117647
**60**	16	16	0	1	1	15	0.0625	0.0625	0.9375	0.9375
**61**	28	31	3	3	0	28	0.09677419	0	1	0.90322581
**62**	27	32	5	5	0	27	0.15625	0	1	0.84375
**63**	21	23	2	2	0	21	0.08695652	0	1	0.91304348
**64**	7	8	1	1	0	7	0.125	0	1	0.875
**65**	13	13	0	0	0	13	0	0	1	1
**66**	14	14	0	0	0	14	0	0	1	1
**67**	11	12	1	1	0	11	0.08333333	0	1	0.91666667
**68**	21	22	1	3	2	19	0.13636364	0.0952381	0.9047619	0.86363636
**69**	18	20	2	3	1	17	0.15	0.05555556	0.94444444	0.85
**70**	13	14	1	1	0	13	0.07142857	0	1	0.92857143
**71**	22	21	1	3	4	18	0.14285714	0.18181818	0.81818182	0.85714286
**72**	23	26	3	4	1	22	0.15384615	0.04347826	0.95652174	0.84615385
**73**	25	27	2	3	1	24	0.11111111	0.04	0.96	0.88888889
**74**	13	17	4	4	0	13	0.23529412	0	1	0.76470588
**75**	21	26	5	5	0	21	0.19230769	0	1	0.80769231
**76**	16	17	1	2	1	15	0.11764706	0.0625	0.9375	0.88235294
**77**	14	15	1	2	1	13	0.13333333	0.07142857	0.92857143	0.86666667
**78**	10	10	0	1	1	9	0.1	0.1	0.9	0.9
**79**	17	17	0	1	1	16	0.05882353	0.05882353	0.94117647	0.94117647
**80**	12	12	0	2	2	10	0.16666667	0.16666667	0.83333333	0.83333333
**81**	11	9	2	0	2	9	0	0.18181818	0.81818182	1
**82**	16	17	1	2	1	15	0.11764706	0.0625	0.9375	0.88235294
**83**	13	14	1	3	2	11	0.21428571	0.15384615	0.84615385	0.78571429
**84**	17	17	0	4	4	13	0.23529412	0.23529412	0.76470588	0.76470588
**85**	22	24	2	2	0	22	0.08333333	0	1	0.91666667
**86**	31	34	3	6	3	28	0.17647059	0.09677419	0.90322581	0.82352941
**87**	20	19	1	2	3	17	0.10526316	0.15	0.85	0.89473684
**88**	16	18	2	4	2	14	0.22222222	0.125	0.875	0.77777778
**89**	16	17	1	2	1	15	0.11764706	0.0625	0.9375	0.88235294
**90**	20	20	0	3	3	17	0.15	0.15	0.85	0.85

**91**	17	27	10	10	0	17	0.37037037	0	1	0.62962963
**92**	23	43	20	20	0	23	0.46511628	0	1	0.53488372
**93**	19	29	10	10	0	19	0.34482759	0	1	0.65517241
**94**	15	17	2	5	3	12	0.29411765	0.2	0.8	0.70588235
**95**	14	11	3	2	5	9	0.18181818	0.35714286	0.64285714	0.81818182
**96**	14	16	2	2	0	14	0.125	0	1	0.875
**97**	18	19	1	1	0	18	0.05263158	0	1	0.94736842
**98**	12	14	2	2	0	12	0.14285714	0	1	0.85714286
**99**	14	36	22	22	0	14	0.61111111	0	1	0.38888889
**100**	12	13	1	1	0	12	0.07692308	0	1	0.92307692
**Average**				**4.46153846**	**1.34615385**	**15.1923077**	**0.18675825**	**0.0859344**	**0.9140656**	**0.81324175**
**Sum**	**1602**	**1872**								

			FindMyCells						
			In number of cells					Metrics	
Recall	F-1 score	Detection accuracy	Total N of cells	Absolute diffrence in N of cells	False +	False -	True +	False +	False -
1	0.875	0.777777778	17		5	6	12	0.29411765	0.33333333
1	0.72727273	0.571428571	17		5	2	12	0.29411765	0.14285714
1	0.90322581	0.823529412	19		4	2	15	0.21052632	0.11764706
1	0.96969697	0.941176471	20		4	1	16	0.2	0.05882353
0.95833333	0.88461538	0.793103448	22		2	8	20	0.09090909	0.28571429
1	0.93333333	0.875	15		3	4	12	0.2	0.25
1	0.89361702	0.807692308	23		1	4	22	0.04347826	0.15384615
1	0.88888889	0.8	10		1	1	9	0.1	0.1
1	0.94117647	0.888888889	18		3	3	15	0.16666667	0.16666667
1	0.84210526	0.727272727	10		2	3	8	0.2	0.27272727
1	0.96774194	0.9375	14		1	3	13	0.07142857	0.1875
0.86666667	0.89655172	0.8125	14		4	4	10	0.28571429	0.28571429
1	0.82758621	0.705882353	17		0	0	17	0	0
0.92857143	0.76470588	0.619047619	11		2	11	9	0.18181818	0.55
1	0.5	0.333333333	8		1	5	7	0.125	0.41666667
1	0.83333333	0.714285714	17		2	6	15	0.11764706	0.28571429
1	0.76190476	0.615384615	16		4	1	12	0.25	0.07692308
1	0.66666667	0.5	21		4	1	17	0.19047619	0.05555556
1	0.8	0.666666667	13		3	2	10	0.23076923	0.16666667
1	0.86956522	0.769230769	13		2	2	11	0.15384615	0.15384615
1	0.6	0.428571429	11		2	5	9	0.18181818	0.35714286
1	0.85714286	0.75	15		6	3	9	0.4	0.25
1	0.90909091	0.833333333	18		2	2	16	0.11111111	0.11111111
1	0.94444444	0.894736842	19		2	2	17	0.10526316	0.10526316
1	0.82051282	0.695652174	27		7	3	20	0.25925926	0.13043478
1	0.51851852	0.35	13		3	10	10	0.23076923	0.5

1	0.82352941	0.7	11	4	3	7	0.36363636	0.3
0.94444444	0.89473684	0.80952381	22	4	2	18	0.18181818	0.1
1	0.92857143	0.866666667	16	2	1	14	0.125	0.06666667
1	0.95	0.904761905	17	1	5	16	0.05882353	0.23809524
1	1	1	11	2	0	9	0.18181818	0
1	1	1	15	3	2	12	0.2	0.14285714
0.95238095	0.93023256	0.869565217	22	3	3	19	0.13636364	0.13636364
1	0.9	0.818181818	15	0	7	15	0	0.31818182
0.96153846	0.89285714	0.806451613	24	0	6	24	0	0.2
0.95652174	0.91666667	0.846153846	26	5	4	21	0.19230769	0.16
0.9	0.87804878	0.782608696	17	1	5	16	0.05882353	0.23809524
0.95	0.9047619	0.826086957	23	3	2	20	0.13043478	0.09090909
0.85714286	0.8372093	0.72	22	3	3	19	0.13636364	0.13636364
1	0.94545455	0.896551724	30	2	1	28	0.06666667	0.03448276
0.89473684	0.89473684	0.80952381	22	5	2	17	0.22727273	0.10526316
0.94444444	0.94444444	0.894736842	16	2	4	14	0.125	0.22222222
1	0.95454545	0.913043478	23	0	0	23	0	0
0.92857143	0.92857143	0.866666667	13	3	4	10	0.23076923	0.28571429
0.66666667	0.66666667	0.5	15	5	2	10	0.33333333	0.16666667
0.90909091	0.93023256	0.869565217	21	3	3	18	0.14285714	0.14285714
1	0.85714286	0.75	6	3	1	3	0.5	0.25
0.76470588	0.76470588	0.619047619	14	2	5	12	0.14285714	0.29411765
0.94444444	0.91891892	0.85	18	3	4	15	0.16666667	0.21052632
0.92857143	0.86666667	0.764705882	16	4	4	12	0.25	0.25
0.92857143	0.86666667	0.764705882	18	4	2	14	0.22222222	0.125
1	1	1	10	2	2	8	0.2	0.2
0.90909091	0.8	0.666666667	11	2	5	9	0.18181818	0.35714286
0.83333333	0.83333333	0.714285714	14	3	1	11	0.21428571	0.08333333
1	0.95081967	0.90625	28	3	7	25	0.10714286	0.21875
0.95833333	0.93877551	0.884615385	23	6	8	17	0.26086957	0.32
1	0.87878788	0.783783784	36	3	4	33	0.08333333	0.10810811
1	0.94736842	0.9	21	3	2	18	0.14285714	0.1

0.94117647	0.94117647	0.888888889	11	2	8	9	0.18181818	0.47058824
0.9375	0.9375	0.882352941	18	4	2	14	0.22222222	0.125
1	0.94915254	0.903225806	30	5	6	25	0.16666667	0.19354839
1	0.91525424	0.84375	20	3	15	17	0.15	0.46875
1	0.95454545	0.913043478	24	3	2	21	0.125	0.08695652
1	0.93333333	0.875	11	5	2	6	0.45454545	0.25
1	1	1	12	2	3	10	0.16666667	0.23076923
1	1	1	17	5	2	12	0.29411765	0.14285714
1	0.95652174	0.916666667	12	4	4	8	0.33333333	0.33333333
0.9047619	0.88372093	0.791666667	22	0	0	22	0	0
0.94444444	0.89473684	0.80952381	18	2	4	16	0.11111111	0.2
1	0.96296296	0.928571429	8	1	7	7	0.125	0.5
0.81818182	0.8372093	0.72	13	3	11	10	0.23076923	0.52380952
0.95652174	0.89795918	0.814814815	19	3	10	16	0.15789474	0.38461538
0.96	0.92307692	0.857142857	25	2	4	23	0.08	0.14814815
1	0.86666667	0.764705882	17	3	3	14	0.17647059	0.17647059
1	0.89361702	0.807692308	23	2	5	21	0.08695652	0.19230769
0.9375	0.90909091	0.833333333	16	2	3	14	0.125	0.17647059
0.92857143	0.89655172	0.8125	13	2	4	11	0.15384615	0.26666667
0.9	0.9	0.818181818	10	2	2	8	0.2	0.2
0.94117647	0.94117647	0.888888889	21	4	0	17	0.19047619	0
0.83333333	0.83333333	0.714285714	11	1	2	10	0.09090909	0.16666667
0.81818182	0.9	0.818181818	10	2	1	8	0.2	0.11111111
0.9375	0.90909091	0.833333333	13	0	4	13	0	0.23529412
0.84615385	0.81481481	0.6875	12	2	4	10	0.16666667	0.28571429
0.76470588	0.76470588	0.619047619	18	7	6	11	0.38888889	0.35294118
1	0.95652174	0.916666667	24	2	2	22	0.08333333	0.08333333
0.90322581	0.86153846	0.756756757	27	2	9	25	0.07407407	0.26470588
0.85	0.87179487	0.772727273	12	0	7	12	0	0.36842105
0.875	0.82352941	0.7	16	0	2	16	0	0.11111111
0.9375	0.90909091	0.833333333	16	2	3	14	0.125	0.17647059
0.85	0.85	0.739130435	18	2	4	16	0.11111111	0.2

1	0.77272727	0.62962963	20	1	8	19	0.05	0.2962963
1	0.6969697	0.534883721	28	3	18	25	0.10714286	0.41860465
1	0.79166667	0.655172414	29	0	0	29	0	0
0.8	0.75	0.6	13	1	5	12	0.07692308	0.29411765
0.64285714	0.72	0.5625	15	5	1	10	0.33333333	0.09090909
1	0.93333333	0.875	18	3	1	15	0.16666667	0.0625
1	0.97297297	0.947368421	20	1	0	19	0.05	0
1	0.92307692	0.857142857	11	6	9	5	0.54545455	0.64285714
1	0.56	0.388888889	22	3	17	19	0.13636364	0.47222222
1	0.96	0.923076923	9	1	5	8	0.11111111	0.38461538
**0.9140656**	**0.85060013**	**0.750970083**		**2.15384615**	**4.69230769**	**14.9615385**	**0.13743297**	**0.22512833**
			**1736**					

							ilastik		
							In number of cells		
True +	Precision	Recall	F-1 score	Detection accuracy	Total N of cells	Absolute diffrence in N of cells	False +	False -	True +
0.66666667	0.70588235	0.66666667	0.68571429	0.52173913	35		18	1	17
0.85714286	0.70588235	0.85714286	0.77419355	0.631578947	37		25	2	12
0.88235294	0.78947368	0.88235294	0.83333333	0.714285714	53		38	2	15
0.94117647	0.8	0.94117647	0.86486486	0.761904762	44		31	4	13
0.71428571	0.90909091	0.71428571	0.8	0.666666667	55		32	5	23
0.75	0.8	0.75	0.77419355	0.631578947	35		23	4	12
0.84615385	0.95652174	0.84615385	0.89795918	0.814814815	29		15	12	14
0.9	0.9	0.9	0.9	0.818181818	36		32	6	4
0.83333333	0.83333333	0.83333333	0.83333333	0.714285714	19		12	11	7
0.72727273	0.8	0.72727273	0.76190476	0.615384615	23		16	4	7
0.8125	0.92857143	0.8125	0.86666667	0.764705882	20		18	14	2
0.71428571	0.71428571	0.71428571	0.71428571	0.555555556	36		29	7	7
1	1	1	1	1	15		14	16	1
0.45	0.81818182	0.45	0.58064516	0.409090909	13		10	17	3
0.58333333	0.875	0.58333333	0.7	0.538461538	71		66	7	5
0.71428571	0.88235294	0.71428571	0.78947368	0.652173913	60		43	4	17
0.92307692	0.75	0.92307692	0.82758621	0.705882353	37		28	4	9
0.94444444	0.80952381	0.94444444	0.87179487	0.772727273	31		16	3	15
0.83333333	0.76923077	0.83333333	0.8	0.666666667	19		16	9	3
0.84615385	0.84615385	0.84615385	0.84615385	0.733333333	31		23	5	8
0.64285714	0.81818182	0.64285714	0.72	0.5625	27		16	3	11
0.75	0.6	0.75	0.66666667	0.5	48		39	3	9
0.88888889	0.88888889	0.88888889	0.88888889	0.8	48		34	4	14
0.89473684	0.89473684	0.89473684	0.89473684	0.80952381	81		64	2	17
0.86956522	0.74074074	0.86956522	0.8	0.666666667	89		66	0	23
0.5	0.76923077	0.5	0.60606061	0.434782609	91		71	0	20

0.7	0.63636364	0.7	0.66666667	0.5	63	53	0	10
0.9	0.81818182	0.9	0.85714286	0.75	60	43	3	17
0.93333333	0.875	0.93333333	0.90322581	0.823529412	58	45	2	13
0.76190476	0.94117647	0.76190476	0.84210526	0.727272727	61	40	0	21
1	0.81818182	1	0.9	0.818181818	42	37	4	5
0.85714286	0.8	0.85714286	0.82758621	0.705882353	55	44	3	11
0.86363636	0.86363636	0.86363636	0.86363636	0.76	44	30	8	14
0.68181818	1	0.68181818	0.81081081	0.681818182	49	34	7	15
0.8	1	0.8	0.88888889	0.8	49	31	12	18
0.84	0.80769231	0.84	0.82352941	0.7	66	41	0	25
0.76190476	0.94117647	0.76190476	0.84210526	0.727272727	66	50	5	16
0.90909091	0.86956522	0.90909091	0.88888889	0.8	103	81	0	22
0.86363636	0.86363636	0.86363636	0.86363636	0.76	80	58	0	22
0.96551724	0.93333333	0.96551724	0.94915254	0.903225806	69	42	2	27
0.89473684	0.77272727	0.89473684	0.82926829	0.708333333	61	43	1	18
0.77777778	0.875	0.77777778	0.82352941	0.7	74	56	0	18
1	1	1	1	1	69	48	2	21
0.71428571	0.76923077	0.71428571	0.74074074	0.588235294	113	99	0	14
0.83333333	0.66666667	0.83333333	0.74074074	0.588235294	85	73	0	12
0.85714286	0.85714286	0.85714286	0.85714286	0.75	111	93	3	18
0.75	0.5	0.75	0.6	0.428571429	71	69	2	2
0.70588235	0.85714286	0.70588235	0.77419355	0.631578947	56	48	9	8
0.78947368	0.83333333	0.78947368	0.81081081	0.681818182	43	30	6	13
0.75	0.75	0.75	0.75	0.6	42	34	8	8
0.875	0.77777778	0.875	0.82352941	0.7	61	48	3	13
0.8	0.8	0.8	0.8	0.666666667	21	16	5	5
0.64285714	0.81818182	0.64285714	0.72	0.5625	36	22	0	14
0.91666667	0.78571429	0.91666667	0.84615385	0.733333333	8	5	9	3
0.78125	0.89285714	0.78125	0.83333333	0.714285714	53	24	3	29
0.68	0.73913043	0.68	0.70833333	0.548387097	37	19	7	18
0.89189189	0.91666667	0.89189189	0.90410959	0.825	54	24	7	30
0.9	0.85714286	0.9	0.87804878	0.782608696	46	35	9	11

0.52941176	0.81818182	0.52941176	0.64285714	0.473684211	33	26	10	7
0.875	0.77777778	0.875	0.82352941	0.7	19	10	7	9
0.80645161	0.83333333	0.80645161	0.81967213	0.694444444	68	44	7	24
0.53125	0.85	0.53125	0.65384615	0.485714286	67	45	10	22
0.91304348	0.875	0.91304348	0.89361702	0.807692308	30	18	11	12
0.75	0.54545455	0.75	0.63157895	0.461538462	6	4	6	2
0.76923077	0.83333333	0.76923077	0.8	0.666666667	33	30	10	3
0.85714286	0.70588235	0.85714286	0.77419355	0.631578947	39	33	8	6
0.66666667	0.66666667	0.66666667	0.66666667	0.5	31	25	6	6
1	1	1	1	1	12	9	19	3
0.8	0.88888889	0.8	0.84210526	0.727272727	44	40	16	4
0.5	0.875	0.5	0.63636364	0.466666667	61	55	8	6
0.47619048	0.76923077	0.47619048	0.58823529	0.416666667	11	8	18	3
0.61538462	0.84210526	0.61538462	0.71111111	0.551724138	79	53	0	26
0.85185185	0.92	0.85185185	0.88461538	0.793103448	64	41	4	23
0.82352941	0.82352941	0.82352941	0.82352941	0.7	70	57	4	13
0.80769231	0.91304348	0.80769231	0.85714286	0.75	38	17	5	21
0.82352941	0.875	0.82352941	0.84848485	0.736842105	68	53	2	15
0.73333333	0.84615385	0.73333333	0.78571429	0.647058824	38	25	2	13
0.8	0.8	0.8	0.8	0.666666667	80	74	4	6
1	0.80952381	1	0.89473684	0.80952381	19	7	5	12
0.83333333	0.90909091	0.83333333	0.86956522	0.769230769	60	58	10	2
0.88888889	0.8	0.88888889	0.84210526	0.727272727	215	212	6	3
0.76470588	1	0.76470588	0.86666667	0.764705882	69	64	12	5
0.71428571	0.83333333	0.71428571	0.76923077	0.625	34	24	4	10
0.64705882	0.61111111	0.64705882	0.62857143	0.458333333	41	31	7	10
0.91666667	0.91666667	0.91666667	0.91666667	0.846153846	38	18	4	20
0.73529412	0.92592593	0.73529412	0.81967213	0.694444444	49	28	13	21
0.63157895	1	0.63157895	0.77419355	0.631578947	51	33	1	18
0.88888889	1	0.88888889	0.94117647	0.888888889	71	55	2	16
0.82352941	0.875	0.82352941	0.84848485	0.736842105	33	23	7	10
0.8	0.88888889	0.8	0.84210526	0.727272727	56	47	11	9

0.7037037	0.95	0.7037037	0.80851064	0.678571429	61	37	3	24
0.58139535	0.89285714	0.58139535	0.70422535	0.543478261	101	66	8	35
1	1	1	1	1	54	30	5	24
0.70588235	0.92307692	0.70588235	0.8	0.666666667	33	24	8	9
0.90909091	0.66666667	0.90909091	0.76923077	0.625	25	22	8	3
0.9375	0.83333333	0.9375	0.88235294	0.789473684	30	19	5	11
1	0.95	1	0.97435897	0.95	36	21	4	15
0.35714286	0.45454545	0.35714286	0.4	0.25	87	78	5	9
0.52777778	0.86363636	0.52777778	0.65517241	0.487179487	84	49	1	35
0.61538462	0.88888889	0.61538462	0.72727273	0.571428571	33	23	3	10
**0.77487167**	**0.86256703**	**0.77487167**	**0.8086785**	**0.693908199**		**43.7692308**	**5.57692308**	**14.0769231**
					**5160**			

T

Metrics									In
False +	False -	True +	Precision	Recall	F-1 score	Detection accuracy	Total N of cells	Absolute diffrence in N of cells	False +
0.51428571	0.05555556	0.94444444	0.48571429	0.94444444	0.64150943	0.472222222	12	2	3
0.67567568	0.14285714	0.85714286	0.32432432	0.85714286	0.47058824	0.307692308	18	10	10
0.71698113	0.11764706	0.88235294	0.28301887	0.88235294	0.42857143	0.272727273	27	13	12
0.70454545	0.23529412	0.76470588	0.29545455	0.76470588	0.42622951	0.270833333	22	6	13
0.58181818	0.17857143	0.82142857	0.41818182	0.82142857	0.55421687	0.383333333	22	2	10
0.65714286	0.25	0.75	0.34285714	0.75	0.47058824	0.307692308	17	3	10
0.51724138	0.46153846	0.53846154	0.48275862	0.53846154	0.50909091	0.341463415	13	8	3
0.88888889	0.6	0.4	0.11111111	0.4	0.17391304	0.095238095	5	3	2
0.63157895	0.61111111	0.38888889	0.36842105	0.38888889	0.37837838	0.233333333	3	13	1
0.69565217	0.36363636	0.63636364	0.30434783	0.63636364	0.41176471	0.259259259	4	4	1
0.9	0.875	0.125	0.1	0.125	0.11111111	0.058823529	2	13	1
0.80555556	0.5	0.5	0.19444444	0.5	0.28	0.162790698	3	12	1
0.93333333	0.94117647	0.05882353	0.06666667	0.05882353	0.0625	0.032258065	1	11	1
0.76923077	0.85	0.15	0.23076923	0.15	0.18181818	0.1	1	13	1
0.92957746	0.58333333	0.41666667	0.07042254	0.41666667	0.12048193	0.064102564	1	3	1
0.71666667	0.19047619	0.80952381	0.28333333	0.80952381	0.41975309	0.265625	16	1	10
0.75675676	0.30769231	0.69230769	0.24324324	0.69230769	0.36	0.219512195	22	14	14
0.51612903	0.16666667	0.83333333	0.48387097	0.83333333	0.6122449	0.441176471	18	9	7
0.84210526	0.75	0.25	0.15789474	0.25	0.19354839	0.107142857	20	12	16
0.74193548	0.38461538	0.61538462	0.25806452	0.61538462	0.36363636	0.222222222	14	4	6
0.59259259	0.21428571	0.78571429	0.40740741	0.78571429	0.53658537	0.366666667	15	9	8
0.8125	0.25	0.75	0.1875	0.75	0.3	0.176470588	24	15	17
0.70833333	0.22222222	0.77777778	0.29166667	0.77777778	0.42424242	0.269230769	29	14	18
0.79012346	0.10526316	0.89473684	0.20987654	0.89473684	0.34	0.204819277	39	22	26
0.74157303	0	1	0.25842697	1	0.41071429	0.258426966	56	40	35
0.78021978	0	1	0.21978022	1	0.36036036	0.21978022	57	50	41

0.84126984	0	1	0.15873016	1	0.2739726	0.158730159	35	28	25
0.71666667	0.15	0.85	0.28333333	0.85	0.425	0.26984127	39	21	22
0.77586207	0.13333333	0.86666667	0.22413793	0.86666667	0.35616438	0.216666667	30	17	18
0.6557377	0	1	0.3442623	1	0.51219512	0.344262295	28	9	15
0.88095238	0.44444444	0.55555556	0.11904762	0.55555556	0.19607843	0.108695652	11	2	7
0.8	0.21428571	0.78571429	0.2	0.78571429	0.31884058	0.189655172	29	15	20
0.68181818	0.36363636	0.63636364	0.31818182	0.63636364	0.42424242	0.269230769	22	1	12
0.69387755	0.31818182	0.68181818	0.30612245	0.68181818	0.42253521	0.267857143	33	15	19
0.63265306	0.4	0.6	0.36734694	0.6	0.4556962	0.295081967	29	3	14
0.62121212	0	1	0.37878788	1	0.54945055	0.378787879	30	7	10
0.75757576	0.23809524	0.76190476	0.24242424	0.76190476	0.36781609	0.225352113	24	4	15
0.78640777	0	1	0.21359223	1	0.352	0.213592233	28	8	14
0.725	0	1	0.275	1	0.43137255	0.275	27	6	11
0.60869565	0.06896552	0.93103448	0.39130435	0.93103448	0.55102041	0.38028169	37	11	13
0.70491803	0.05263158	0.94736842	0.29508197	0.94736842	0.45	0.290322581	40	21	29
0.75675676	0	1	0.24324324	1	0.39130435	0.243243243	40	22	27
0.69565217	0.08695652	0.91304348	0.30434783	0.91304348	0.45652174	0.295774648	37	16	21
0.87610619	0	1	0.12389381	1	0.22047244	0.123893805	44	30	32
0.85882353	0	1	0.14117647	1	0.24742268	0.141176471	17	5	11
0.83783784	0.14285714	0.85714286	0.16216216	0.85714286	0.27272727	0.157894737	38	16	29
0.97183099	0.5	0.5	0.02816901	0.5	0.05333333	0.02739726	16	13	15
0.85714286	0.52941176	0.47058824	0.14285714	0.47058824	0.21917808	0.123076923	21	4	18
0.69767442	0.31578947	0.68421053	0.30232558	0.68421053	0.41935484	0.265306122	12	6	7
0.80952381	0.5	0.5	0.19047619	0.5	0.27586207	0.16	14	0	11
0.78688525	0.1875	0.8125	0.21311475	0.8125	0.33766234	0.203125	22	8	13
0.76190476	0.5	0.5	0.23809524	0.5	0.32258065	0.192307692	6	4	1
0.61111111	0	1	0.38888889	1	0.56	0.388888889	14	3	6
0.625	0.75	0.25	0.375	0.25	0.3	0.176470588	1	11	0
0.45283019	0.09375	0.90625	0.54716981	0.90625	0.68235294	0.517857143	19	10	8
0.51351351	0.28	0.72	0.48648649	0.72	0.58064516	0.409090909	9	15	2
0.44444444	0.18918919	0.81081081	0.55555556	0.81081081	0.65934066	0.491803279	18	11	1
0.76086957	0.45	0.55	0.23913043	0.55	0.33333333	0.2	5	13	1

0.78787879	0.58823529	0.41176471	0.21212121	0.41176471	0.28	0.162790698	8	9	7
0.52631579	0.4375	0.5625	0.47368421	0.5625	0.51428571	0.346153846	2	14	1
0.64705882	0.22580645	0.77419355	0.35294118	0.77419355	0.48484848	0.32	23	5	8
0.67164179	0.3125	0.6875	0.32835821	0.6875	0.44444444	0.285714286	21	6	10
0.6	0.47826087	0.52173913	0.4	0.52173913	0.45283019	0.292682927	3	18	0
0.66666667	0.75	0.25	0.33333333	0.25	0.28571429	0.166666667	2	5	1
0.90909091	0.76923077	0.23076923	0.09090909	0.23076923	0.13043478	0.069767442	1	12	1
0.84615385	0.57142857	0.42857143	0.15384615	0.42857143	0.22641509	0.127659574	3	11	1
0.80645161	0.5	0.5	0.19354839	0.5	0.27906977	0.162162162	4	7	1
0.75	0.86363636	0.13636364	0.25	0.13636364	0.17647059	0.096774194	4	17	2
0.90909091	0.8	0.2	0.09090909	0.2	0.125	0.066666667	1	17	0
0.90163934	0.57142857	0.42857143	0.09836066	0.42857143	0.16	0.086956522	1	12	1
0.72727273	0.85714286	0.14285714	0.27272727	0.14285714	0.1875	0.103448276	1	21	0
0.67088608	0	1	0.32911392	1	0.4952381	0.329113924	38	15	17
0.640625	0.14814815	0.85185185	0.359375	0.85185185	0.50549451	0.338235294	33	8	16
0.81428571	0.23529412	0.76470588	0.18571429	0.76470588	0.29885057	0.175675676	33	20	20
0.44736842	0.19230769	0.80769231	0.55263158	0.80769231	0.65625	0.488372093	23	2	6
0.77941176	0.11764706	0.88235294	0.22058824	0.88235294	0.35294118	0.214285714	27	11	17
0.65789474	0.13333333	0.86666667	0.34210526	0.86666667	0.49056604	0.325	13	1	5
0.925	0.4	0.6	0.075	0.6	0.13333333	0.071428571	7	3	1
0.36842105	0.29411765	0.70588235	0.63157895	0.70588235	0.66666667	0.5	6	11	1
0.96666667	0.83333333	0.16666667	0.03333333	0.16666667	0.05555556	0.028571429	4	8	3
0.98604651	0.66666667	0.33333333	0.01395349	0.33333333	0.02678571	0.013574661	3	8	3
0.92753623	0.70588235	0.29411765	0.07246377	0.29411765	0.11627907	0.061728395	1	15	0
0.70588235	0.28571429	0.71428571	0.29411765	0.71428571	0.41666667	0.263157895	8	5	4
0.75609756	0.41176471	0.58823529	0.24390244	0.58823529	0.34482759	0.208333333	12	5	3
0.47368421	0.16666667	0.83333333	0.52631579	0.83333333	0.64516129	0.476190476	13	9	4
0.57142857	0.38235294	0.61764706	0.42857143	0.61764706	0.5060241	0.338709677	16	15	7
0.64705882	0.05263158	0.94736842	0.35294118	0.94736842	0	0.346153846	27	7	10
0.77464789	0.11111111	0.88888889	0.22535211	0.88888889	0.35955056	0.219178082	31	15	16
0.6969697	0.41176471	0.58823529	0.3030303	0.58823529	0.4	0.25	9	7	2
0.83928571	0.55	0.45	0.16071429	0.45	0.23684211	0.134328358	11	9	4

0.60655738	0.11111111	0.88888889	0.39344262	0.88888889	0.54545455	0.375	28	11	11
0.65346535	0.18604651	0.81395349	0.34653465	0.81395349	0.48611111	0.321100917	35	12	13
0.55555556	0.17241379	0.82758621	0.44444444	0.82758621	0.57831325	0.406779661	27	8	8
0.72727273	0.47058824	0.52941176	0.27272727	0.52941176	0.36	0.219512195	5	10	0
0.88	0.72727273	0.27272727	0.12	0.27272727	0.16666667	0.090909091	1	13	1
0.63333333	0.3125	0.6875	0.36666667	0.6875	0.47826087	0.314285714	5	9	0
0.58333333	0.21052632	0.78947368	0.41666667	0.78947368	0.54545455	0.375	16	2	4
0.89655172	0.35714286	0.64285714	0.10344828	0.64285714	0.17821782	0.097826087	36	24	26
0.58333333	0.02777778	0.97222222	0.41666667	0.97222222	0.58333333	0.411764706	43	29	14
0.6969697	0.23076923	0.76923077	0.3030303	0.76923077	0.43478261	0.277777778	8	4	3
**0.70537587**	**0.32774779**	**0.67225221**	**0.29462413**	**0.67225221**	**0.37554018**	**0.262652642**			**6.38461538**
							**1829**		

Threshold based method

number of cells			Metrics						
False -	True +	False +	False -	True +	Precision	Recall	F-1 score	Detection accuracy	Total N of cells
9	9	0.25	0.5	0.5	0.75	0.5	0.6	0.428571429	12
6	8	0.55555556	0.42857143	0.57142857	0.44444444	0.57142857	0.5	0.333333333	9
2	15	0.44444444	0.11764706	0.88235294	0.55555556	0.88235294	0.68181818	0.517241379	5
8	9	0.59090909	0.47058824	0.52941176	0.40909091	0.52941176	0	0.3	0
16	12	0.45454545	0.57142857	0.42857143	0.54545455	0.42857143	0	0.315789474	19
9	7	0.58823529	0.5625	0.4375	0.41176471	0.4375	0.42424242	0.269230769	0
16	10	0.23076923	0.61538462	0.38461538	0.76923077	0.38461538	0.51282051	0.344827586	0
7	3	0.4	0.7	0.3	0.6	0.3	0.4	0.25	1
16	2	0.33333333	0.88888889	0.11111111	0.66666667	0.11111111	0.19047619	0.105263158	3
8	3	0.25	0.72727273	0.27272727	0.75	0.27272727	0	0.25	1
15	1	0.5	0.9375	0.0625	0.5	0.0625	0.11111111	0.058823529	4
12	2	0.33333333	0.85714286	0.14285714	0.66666667	0.14285714	0.23529412	0.133333333	2
17	0	1	1	0	0	0	0	0	1
20	0	1	1	0	0	0	0	0	1
12	0	1	1	0	0	0	0	0	1
15	6	0.625	0.71428571	0.28571429	0.375	0.28571429	0.32432432	0.193548387	2
5	8	0.63636364	0.38461538	0.61538462	0.36363636	0.61538462	0.45714286	0.296296296	2
7	11	0.38888889	0.38888889	0.61111111	0.61111111	0.61111111	0.61111111	0.44	8
8	4	0.8	0.66666667	0.33333333	0.2	0.33333333	0.25	0.142857143	3
5	8	0.42857143	0.38461538	0.61538462	0.57142857	0.61538462	0.59259259	0.421052632	24
7	7	0.53333333	0.5	0.5	0.46666667	0.5	0.48275862	0.318181818	3
5	7	0.70833333	0.41666667	0.58333333	0.29166667	0.58333333	0.38888889	0.24137931	0
7	11	0.62068966	0.38888889	0.61111111	0.37931034	0.61111111	0.46808511	0.305555556	0
6	13	0.66666667	0.31578947	0.68421053	0.33333333	0.68421053	0.44827586	0.288888889	0
2	21	0.625	0.08695652	0.91304348	0.375	0.91304348	0.53164557	0.362068966	6
4	16	0.71929825	0.2	0.8	0.28070175	0.8	0.41558442	0.262295082	0

0	10	0.71428571	0	1	0.28571429	1	0.44444444	0.285714286	0
3	17	0.56410256	0.15	0.85	0.43589744	0.85	0.57627119	0.404761905	0
3	12	0.6	0.2	0.8	0.4	0.8	0.53333333	0.363636364	22
8	13	0.53571429	0.38095238	0.61904762	0.46428571	0.61904762	0.53061224	0.361111111	29
5	4	0.63636364	0.55555556	0.44444444	0.36363636	0.44444444	0.4	0.25	3
5	9	0.68965517	0.35714286	0.64285714	0.31034483	0.64285714	0.41860465	0.264705882	0
12	10	0.54545455	0.54545455	0.45454545	0.45454545	0.45454545	0.45454545	0.294117647	0
8	14	0.57575758	0.36363636	0.63636364	0.42424242	0.63636364	0.50909091	0.341463415	1
15	15	0.48275862	0.5	0.5	0.51724138	0.5	0.50847458	0.340909091	10
5	20	0.33333333	0.2	0.8	0.66666667	0.8	0.72727273	0.571428571	1
12	9	0.625	0.57142857	0.42857143	0.375	0.42857143	0.4	0.25	0
8	14	0.5	0.36363636	0.63636364	0.5	0.63636364	0.56	0.388888889	2
6	16	0.40740741	0.27272727	0.72727273	0.59259259	0.72727273	0.65306122	0.484848485	0
5	24	0.35135135	0.17241379	0.82758621	0.64864865	0.82758621	0.72727273	0.571428571	24
8	11	0.725	0.42105263	0.57894737	0.275	0.57894737	0.37288136	0.229166667	5
5	13	0.675	0.27777778	0.72222222	0.325	0.72222222	0.44827586	0.288888889	1
7	16	0.56756757	0.30434783	0.69565217	0.43243243	0.69565217	0.53333333	0.363636364	0
2	12	0.72727273	0.14285714	0.85714286	0.27272727	0.85714286	0.4137931	0.260869565	1
6	6	0.64705882	0.5	0.5	0.35294118	0.5	0.4137931	0.260869565	1
12	9	0.76315789	0.57142857	0.42857143	0.23684211	0.42857143	0.30508475	0.18	1
3	1	0.9375	0.75	0.25	0.0625	0.25	0	0.052631579	1
14	3	0.85714286	0.82352941	0.17647059	0.14285714	0.17647059	0.15789474	0.085714286	18
14	5	0.58333333	0.73684211	0.26315789	0.41666667	0.26315789	0.32258065	0.192307692	0
13	3	0.78571429	0.8125	0.1875	0.21428571	0.1875	0.2	0.111111111	9
7	9	0.59090909	0.4375	0.5625	0.40909091	0.5625	0.47368421	0.310344828	4
5	5	0.16666667	0.5	0.5	0.83333333	0.5	0.625	0.454545455	1
6	8	0.42857143	0.42857143	0.57142857	0.57142857	0.57142857	0.57142857	0.4	0
11	1	0	0.91666667	0.08333333	1	0.08333333	0.15384615	0.083333333	1
21	11	0.42105263	0.65625	0.34375	0.57894737	0.34375	0.43137255	0.275	23
18	7	0.22222222	0.72	0.28	0.77777778	0.28	0.41176471	0.259259259	5
20	17	0.05555556	0.54054054	0.45945946	0.94444444	0.45945946	0.61818182	0.447368421	18
16	4	0.2	0.8	0.2	0.8	0.2	0	0.19047619	3

16	1	0.875	0.94117647	0.05882353	0.125	0.05882353	0.08	0.041666667	4
15	1	0.5	0.9375	0.0625	0.5	0.0625	0	0.058823529	1
16	15	0.34782609	0.51612903	0.48387097	0.65217391	0.48387097	0.55555556	0.384615385	0
21	11	0.47619048	0.65625	0.34375	0.52380952	0.34375	0	0.261904762	0
20	3	0	0.86956522	0.13043478	1	0.13043478	0	0.130434783	1
7	1	0.5	0.875	0.125	0.5	0.125	0	0.111111111	1
13	0	1	1	0	0	0	#DIV/0!	0	0
12	2	0.33333333	0.85714286	0.14285714	0.66666667	0.14285714	0.23529412	0.133333333	2
9	3	0.25	0.75	0.25	0.75	0.25	0	0.230769231	1
20	2	0.5	0.90909091	0.09090909	0.5	0.09090909	0	0.083333333	2
19	1	0	0.95	0.05	1	0.05	0	0.05	1
14	0	1	1	0	0	0	0	0	1
20	1	0	0.95238095	0.04761905	1	0.04761905	0	0.047619048	1
5	21	0.44736842	0.19230769	0.80769231	0.55263158	0.80769231	0.65625	0.488372093	0
10	17	0.48484848	0.37037037	0.62962963	0.51515152	0.62962963	0	0.395348837	22
4	13	0.60606061	0.23529412	0.76470588	0.39393939	0.76470588	0	0.351351351	1
9	17	0.26086957	0.34615385	0.65384615	0.73913043	0.65384615	0.69387755	0.53125	9
7	10	0.62962963	0.41176471	0.58823529	0.37037037	0.58823529	0.45454545	0.294117647	17
7	8	0.38461538	0.46666667	0.53333333	0.61538462	0.53333333	0.57142857	0.4	13
4	6	0.14285714	0.4	0.6	0.85714286	0.6	0.70588235	0.545454545	1
12	5	0.16666667	0.70588235	0.29411765	0.83333333	0.29411765	0.43478261	0.277777778	2
11	1	0.75	0.91666667	0.08333333	0.25	0.08333333	0.125	0.066666667	1
9	0	1	1	0	0	0	0	0	3
16	1	0	0.94117647	0.05882353	1	0.05882353	0	0.058823529	1
10	4	0.5	0.71428571	0.28571429	0.5	0.28571429	0.36363636	0.222222222	5
8	9	0.25	0.47058824	0.52941176	0.75	0.52941176	0.62068966	0.45	4
15	9	0.30769231	0.625	0.375	0.69230769	0.375	0.48648649	0.321428571	12
25	9	0.4375	0.73529412	0.26470588	0.5625	0.26470588	0.36	0.219512195	2
2	17	0.37037037	0.10526316	0.89473684	0.62962963	0.89473684	0.73913043	0.586206897	0
3	15	0.51612903	0.16666667	0.83333333	0.48387097	0.83333333	0	0.441176471	0
10	7	0.22222222	0.58823529	0.41176471	0.77777778	0.41176471	0.53846154	0.368421053	14
13	7	0.36363636	0.65	0.35	0.63636364	0.35	0.4516129	0.291666667	3

10	17	0.39285714	0.37037037	0.62962963	0.60714286	0.62962963	0.61818182	0.447368421	21
21	22	0.37142857	0.48837209	0.51162791	0.62857143	0.51162791	0.56410256	0.392857143	1
10	19	0.2962963	0.34482759	0.65517241	0.7037037	0.65517241	0.67857143	0.513513514	19
12	5	0	0.70588235	0.29411765	1	0.29411765	0.45454545	0.294117647	1
11	0	1	1	0	0	0	0	0	1
11	5	0	0.6875	0.3125	1	0.3125	0.47619048	0.3125	4
7	12	0.25	0.36842105	0.63157895	0.75	0.63157895	0.68571429	0.52173913	10
4	10	0.72222222	0.28571429	0.71428571	0.27777778	0.71428571	0.4	0.25	14
7	29	0.3255814	0.19444444	0.80555556	0.6744186	0.80555556	0.73417722	0.58	0
8	5	0.375	0.61538462	0.38461538	0.625	0.38461538	0	0.3125	3
**10.0769231**	**9.57692308**	**0.38598363**	**0.55017541**	**0.44982459**	**0.61401637**	**0.44982459**	**0.42911604**	**0.334589234**	
									**490**

ImageJ

	In number of cells			Metrics					
Absolute diffrence in N of cells	False +	False -	True +	False +	False -	True +	Precision	Recall	F-1 score
6	3	9	9	0.25	0.5	0.5	0.75	0.5	0.6
5	3	8	6	0.33333333	0.57142857	0.42857143	0.66666667	0.42857143	0.52173913
12	2	14	3	0.4	0.82352941	0.17647059	0.6	0.17647059	0.27272727
17	0	17	0	0	1	0	0	0	0
9	10	19	9	0.52631579	0.67857143	0.32142857	0.47368421	0.32142857	0
16	0	16	0	0	1	0	0	0	0
26	0	26	0	0	1	0	0	0	0
9	0	9	1	0	0.9	0.1	1	0.1	0.18181818
15	1	16	2	0	0.88888889	0.11111111	0.66666667	0.11111111	0.19047619
10	1	11	0	1	1	0	0	0	0
12	2	14	2	0.5	0.875	0.125	0.5	0.125	0.2
12	0	12	2	0	0.85714286	0.14285714	1	0.14285714	0.25
16	1	17	0	1	1	0	0	0	0
19	1	20	0	1	1	0	0	0	0
11	1	12	0	1	1	0	0	0	0
19	2	21	0	1	1	0	0	0	0
11	0	11	2	0	0.84615385	0.15384615	1	0.15384615	0.26666667
10	3	13	5	0.375	0.72222222	0.27777778	0.625	0.27777778	0.38461538
9	2	11	1	0.66666667	0.91666667	0.08333333	0.33333333	0.08333333	0.13333333
11	13	2	11	0.54166667	0.15384615	0.84615385	0.45833333	0.84615385	0.59459459
11	1	12	2	0.33333333	0.85714286	0.14285714	0.66666667	0.14285714	0.23529412
12	0	12	0	0	1	0	0	0	0
18	0	18	0	0	1	0	0	0	0
19	0	19	0	0	1	0	0	0	0
17	4	21	2	0	0.91304348	0.08695652	0.33333333	0.08695652	0.13793103
20	0	20	0	0	1	0	0	0	0

10	0	10	0	0	1	0	0	0	0
20	0	20	0	0	1	0	0	0	0
7	11	4	11	0	0.26666667	0.73333333	0.5	0.73333333	0.59459459
8	16	8	13	0.55172414	0.38095238	0.61904762	0.44827586	0.61904762	0.52
6	1	7	2	0.33333333	0.77777778	0.22222222	0.66666667	0.22222222	0.33333333
14	0	14	0	0	1	0	0	0	0
22	0	22	0	0	1	0	0	0	0
21	0	21	1	0	0.95454545	0.04545455	1	0.04545455	0.08695652
20	4	24	6	0	0.8	0.2	0.6	0.2	0.3
24	0	24	1	0	0.96	0.04	1	0.04	0.07692308
21	0	21	0	0	1	0	0	0	0
20	1	21	1	0.5	0.95454545	0.04545455	0.5	0.04545455	0.08333333
22	0	22	0	0	1	0	0	0	0
5	7	12	17	0.29166667	0.4137931	0.5862069	0.70833333	0.5862069	0.64150943
14	4	18	1	0	0.94736842	0.05263158	0.2	0.05263158	0.08333333
17	1	18	0	1	1	0	0	0	0
23	0	23	0	0	1	0	0	0	0
13	0	13	1	0	0.92857143	0.07142857	1	0.07142857	0.13333333
11	0	11	1	0	0.91666667	0.08333333	1	0.08333333	0.15384615
20	0	20	1	0	0.95238095	0.04761905	1	0.04761905	0.09090909
3	1	0	4	1	0	1	0.8	1	0
1	15	14	3	0.83333333	0.82352941	0.17647059	0.16666667	0.17647059	0.17142857
19	0	19	0	0	1	0	0	0	0
7	6	13	3	0.66666667	0.8125	0.1875	0.33333333	0.1875	0.24
12	2	14	2	0	0.875	0.125	0.5	0.125	0.2
9	1	10	0	1	1	0	0	0	0
14	0	14	0	0	1	0	0	0	0
11	1	12	0	1	1	0	0	0	0
9	10	19	13	0	0.59375	0.40625	0.56521739	0.40625	0.47272727
20	0	20	5	0	0.8	0.2	1	0.2	0.33333333
19	1	20	17	0.05555556	0.54054054	0.45945946	0.94444444	0.45945946	0.61818182
17	1	18	2	0.33333333	0.9	0.1	0.66666667	0.1	0

13	4	17	0	1	1	0	0	0	#DIV/0!
15	0	15	1	0	0.9375	0.0625	1	0.0625	0
31	0	31	0	0	1	0	0	0	0
32	0	32	0	0	1	0	0	0	0
22	1	23	0	1	1	0	0	0	0
7	1	8	0	1	1	0	0	0	0
13	0	13	0	0	1	0	0	0	0
12	1	13	1	0	0.92857143	0.07142857	0.5	0.07142857	0.125
11	0	11	1	0	0.91666667	0.08333333	1	0.08333333	0
20	2	22	0	1	1	0	0	0	0
19	1	20	0	0	1	0	0	0	0
13	1	14	0	1	1	0	0	0	0
20	1	21	0	1	1	0	0	0	0
26	0	26	0	#DIV/0!	1	0	0	0	0
5	10	15	12	0.45454545	0.55555556	0.44444444	0.54545455	0.44444444	0
16	1	0	17	1	0	1	0.94444444	1	0
17	1	18	8	0.11111111	0.69230769	0.30769231	0.88888889	0.30769231	0.45714286
0	12	12	5	0	0.70588235	0.29411765	0.29411765	0.29411765	0.29411765
2	5	7	8	0.38461538	0.46666667	0.53333333	0.61538462	0.53333333	0.57142857
9	1	0	10	1	0	1	0.90909091	1	0.95238095
15	0	15	2	0	0.88235294	0.11764706	1	0.11764706	0.21052632
11	1	0	12	0	0	1	0.92307692	1	0.96
6	3	9	0	0	1	0	0	0	0
16	1	17	0	1	1	0	0	0	0
9	2	11	3	0.4	0.78571429	0.21428571	0.6	0.21428571	0.31578947
13	2	15	2	0.5	0.88235294	0.11764706	0.5	0.11764706	0.19047619
12	3	15	9	0.25	0.625	0.375	0.75	0.375	0.5
32	2	0	34	1	0	1	0.94444444	1	0.97142857
19	0	19	0	0	1	0	0	0	0
18	0	18	0	0	1	0	0	0	0
3	4	7	10	0.28571429	0.41176471	0.58823529	0.71428571	0.58823529	0.64516129
17	3	20	0	1	1	0	0	0	0

6	8	14	13	0.38095238	0.51851852	0.48148148	0.61904762	0.48148148	0.54166667
42	1	0	43	1	0	1	0.97727273	1	0.98850575
10	5	15	14	0.26315789	0.51724138	0.48275862	0.73684211	0.48275862	0.58333333
16	0	16	1	0	0.94117647	0.05882353	1	0.05882353	0.11111111
10	1	11	0	0	1	0	0	0	0
12	0	12	4	0	0.75	0.25	1	0.25	0.4
9	2	11	8	0.2	0.57894737	0.42105263	0.8	0.42105263	0.55172414
0	9	9	5	0.64285714	0.64285714	0.35714286	0.35714286	0.35714286	0.35714286
36	0	36	0	0	1	0	0	0	0
10	0	10	3	0	0.76923077	0.23076923	1	0.23076923	0
	**2.53846154**	**12.1923077**	**7.46153846**	**0.32378493**	**0.66038512**	**0.33961488**	**0.56267671**	**0.33961488**	**0.36930522**

Detection accuracy
0.428571429
0.352941176
0.157894737
0
0.236842105
0
0
0.1
0.105263158
0
0.111111111
0.142857143
0
0
0
0
0.153846154
0.238095238
0.071428571
0.423076923
0.133333333
0
0
0
0.074074074
0

0
0
0.423076923
0.351351351
0.2
0
0
0.045454545
0.176470588
0.04
0
0.043478261
0
0.472222222
0.043478261
0
0
0.071428571
0.083333333
0.047619048
0.8
0.09375
0
0.136363636
0.111111111
0
0
0
0.30952381
0.2
0.447368421
0.095238095

0
0.0625
0
0
0
0
0
0.066666667
0.083333333
0
0
0
0
0
0.324324324
0.944444444
0.296296296
0.172413793
0.4
0.909090909
0.117647059
0.923076923
0
0
0.1875
0.105263158
0.333333333
0.944444444
0
0
0.476190476
0

0.371428571
0.977272727
0.411764706
0.058823529
0
0.25
0.380952381
0.217391304
0
0.230769231
**0.298602263**
